# Peripheral Dopamine Directly Acts on Insulin-Sensitive Tissues to Regulate Insulin Signaling and Metabolic Function

**DOI:** 10.3389/fphar.2021.713418

**Published:** 2021-09-09

**Authors:** Gabriela Tavares, Fatima. O. Martins, Bernardete. F. Melo, Paulo Matafome, Silvia. V. Conde

**Affiliations:** ^1^ CEDOC, NOVA Medical School, Faculdade de Ciências Médicas, Universidade Nova de Lisboa, Lisboa, Portugal; ^2^ Institute of Clinical and Biomedical Research (iCBR), Faculty of Medicine, University of Coimbra, Coimbra, Portugal; ^3^ Center for Innovative Biomedicine and Biotechnology (CIBB), University of Coimbra, Coimbra, Portugal; ^4^ Clinical-Academic Center of Coimbra, Coimbra, Portugal; ^5^ Coimbra Health School, Instituto Politécnico de Coimbra, Coimbra, Portugal

**Keywords:** dopamine, insulin signaling, glucose metabolism, lipid metabolism, D1 dopamine receptor, D2 dopamine receptor

## Abstract

Dopamine is a key regulator of glucose metabolism in the central nervous system. However, dopamine is also present in the periphery and may have direct effects on insulin-sensitive tissues. Dopamine receptor 2 (D2R) agonist bromocriptine is a FDA-approved drug for type 2 diabetes. Herein, we explored the role of peripheral dopamine and its receptors in regulating glucose uptake and metabolism on insulin-sensitive tissues. Peripheral dopamine effect in [3H]2-deoxyglucose uptake in insulin-sensitive tissues was tested *in vivo* in rats. Direct effects on [3H]2-deoxyglucose uptake, insulin receptor phosphorylation, and regulation of metabolic function were tested ex *vivo* in the liver, soleus muscle, and white and brown adipose tissues. Bromocriptine and the antagonists domperidone, D2R antagonist, and haloperidol, antagonist of both dopamine receptor 1 (D1R) and D2R, were used to disclose dopamine receptors’ involvement.

Peripheral dopamine increases glucose uptake *in vivo*. Ex *vivo,* only dopamine increased glucose uptake in the soleus, while bromocriptine increased it in the liver; the effects were reverted by haloperidol and domperidone, respectively. In adipose tissue, domperidone reverted dopamine- and bromocriptine-mediated potentiation of insulin-induced glucose uptake, but in turn increased the insulin receptor, Akt, AMPK, HSL, ACC, and ACL, phosphorylation. In the soleus muscle, AMPK-phosphorylation increased with bromocriptine and dopamine whose effects were suppressed by domperidone and haloperidol.

In conclusion, peripheral dopamine stimulates glucose uptake with its receptors being differentially involved in glucose uptake in insulin-sensitive tissues. Dopamine also has a role in lipid metabolism in white adipose tissue. Altogether, these results suggest that peripheral modulation of the dopaminergic system should be further evaluated as a putative therapeutic approach for metabolic disorders.

## Introduction

Dopamine is the predominant catecholamine neurotransmitter in the mammalian central nervous system (CNS) regulating neuronal functions such as locomotor activity and movement coordination, cognition, emotion, positive reinforcement, food intake, and endocrine homeostasis (Missale et al., 1998). Dopamine exerts its actions through binding to G protein–coupled receptors, classified in two subfamilies: the dopamine D1-like receptors family (D1R and D5R), which are linked to a stimulatory effect on adenylyl cyclase and therefore increase cyclic adenosine monophosphate (cAMP) levels, and the dopamine D2-like receptors (D2R, D3R, and D4R) that have an inhibitory effect on adenylyl cyclase, leading to a decrease in cAMP levels (Bone et al., 2017; Bucolo et al., 2019).

Outside the CNS, dopamine derived from the synaptic terminals of the peripheral nervous system regulates several processes such as hormone secretion, vascular tone, renal function, and gastrointestinal motility (Asghar et al., 2011; Bucolo et al., 2019), also playing a key role in the central regulation of glucose metabolism and energy balance. Mice lacking whole body D2R are glucose intolerant and exhibit abnormal insulin secretion (Garcia-Tornadú et al., 2010). Moreover, adverse metabolic profiles in mice, such as hyperinsulinemia, weight gain, and glucose intolerance, were observed after dopamine receptors blockade with haloperidol, a dopamine D1R + D2R antagonist, and after administration of some antipsychotic drugs in humans (de Leeuw van Weenen et al., 2010; Garcia-Tornadú et al., 2010; Freyberg and McCarthy, 2017). In agreement with this role, the D2R agonist bromocriptine was shown to improve glycemic control and glucose tolerance in obese type 2 diabetic patients, being approved by the FDA for the treatment of type 2 diabetes. Such effects were described to be associated with increased hypothalamic dopamine levels and inhibition of excessive sympathetic tone within the central nervous system (Via et al., 2010; DeFronzo, 2011; Lopez Vicchi et al., 2016). More recently, we showed that bromocriptine remodels adipose tissue dopaminergic signaling and upregulates catabolic pathways, improving the metabolic profile in type 2 diabetes (Tavares et al., 2021). Such effects may be involved in bromocriptine therapeutic action, given the impaired expression of dopamine receptors in the visceral adipose tissue of insulin-resistant patients, as well as the correlation of dopamine D1R expression with insulin receptors and metabolic mediators (Tavares et al., 2021). Altogether, the findings of Tavares et al. (2021) suggest a possible peripheral action of bromocriptine on glucose metabolism. In fact, several evidences also point toward the role of dopamine in the regulation of peripheral glucose homeostasis and insulin sensitivity and secretion: 1) the dopamine transporter—the vesicular monoamine transporter type 2—was found to be expressed by insulin-producing β cells, but not in glucagon or somatostatin-expressing ones (Ustione et al., 2013; Maffei et al., 2015; Chaudhry et al., 2016; Bucolo et al., 2019); 2) dopamine D2-like receptors are expressed in pancreatic β-cells, and its targeting through exogenous dopamine inhibits glucose-stimulated insulin secretion from the isolated islets (Ustione et al., 2013; Chaudhry et al., 2016, 2016); 3) dopamine is produced by rat adipocytes isolated from mesenteric adipose tissue (Vargovic et al., 2011); and 4) all dopamine receptors, except the D3R, were found to be expressed in human white adipose tissue, involved in the modulation of hormones such as leptin, adiponectin, prolactin, and interleukin-6 (Borcherding et al., 2011). However, more studies are needed to clarify and test the hypothesis that dopamine is a potential modulator of insulin signaling, glucose uptake, and activation of metabolic pathways in the periphery.

In the present study, we evaluated the role of peripheral exogenous dopamine in regulating glucose uptake, as well as its direct effect on glucose uptake, insulin signaling, and lipid metabolic function of insulin-sensitive tissues. Additionally, we characterize the dopamine receptors involved in these actions. We found that exogenous dopamine administration promotes glucose uptake. Moreover, dopamine directly acts on insulin-sensitive tissues through different receptors to regulate glucose uptake and insulin receptor phosphorylation and to modulate the metabolic function, namely, adenosine monophosphate kinase (AMPK) phosphorylation in the liver, adipose tissue, and the skeletal muscle and to regulate lipid metabolism–related proteins in the adipose tissue. Therefore, we postulated that the modulation of peripheral dopaminergic signaling in insulin-sensitive tissues must be further evaluated as a potential therapeutic approach to treat metabolic disorders.

## Materials and Methods

### Ethical Approval

All experimental and care procedures conducted on the animals were approved by the Ethics Committee of Faculdade de Ciencias Médicas|Nova Medical School and by Direccao-Geral de Alimentacao e Veterinária (DGAV), the Portuguese National Authority for Animal Health Animal Care. Principles of laboratory care were followed in accordance with the European Union Directive for Protection of Vertebrates Used for Experimental and Other Scientific Ends (2010/63/EU).

#### Animals

Experiments were performed on 8- to 10-week-old male Wistar rats (200–300 g) obtained from the vivarium of the Faculdade de Ciências Médicas|Nova Medical School, Portugal. Animals were kept under controlled temperature and humidity (21 ± 1°C; 55 ± 10% humidity) with a 12-h light/dark cycle and ad libitum access to food and water.

#### *In vivo* tissue-specific glucose uptake

**FIGURE 1:**
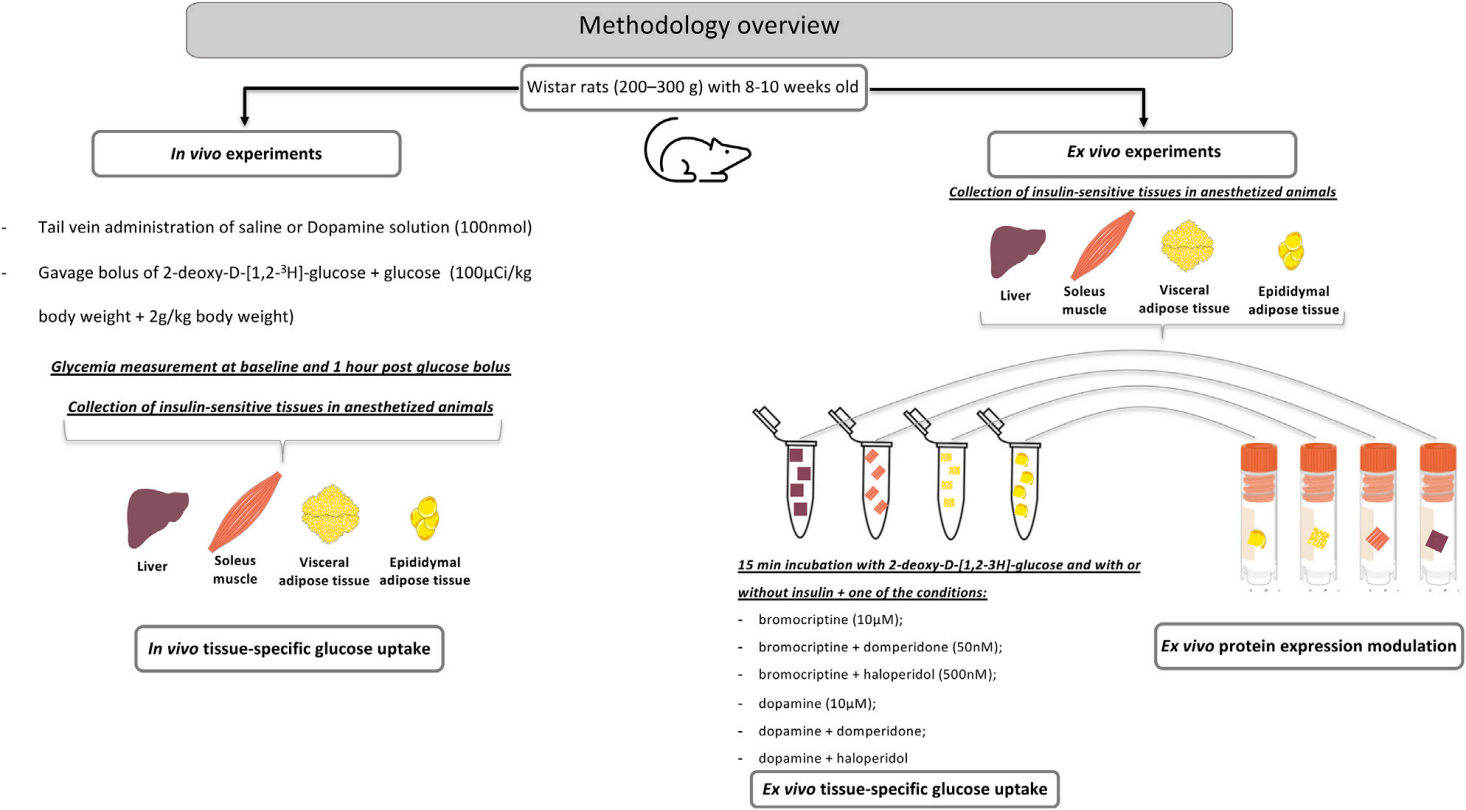
A methodological overview for the *in vivo* and ex vivo experiments aimed to study the role of peripheral dopamine on insulin-sensitive tissues.

Animals were starved overnight and randomly divided into the control group (0.9% NaCl) versus dopamine (Medopa, Medinfar)-injected group (100 nmol). Dopamine or saline were administered in the tail vein in a bolus of 0.1 ml. Immediately after dopamine or saline administration, an oral glucose tolerance test (OGTT) with a bolus of 2-deoxy-D-[1,2–3H]-glucose (2-DG, 1 mCi/ ml; specific activity: 8 Ci/mmol; PerkinElmer, Madrid, Spain) mixed with glucose (100 μ Ci/ kg body weight; 2 g/ kg body weight) was performed by gavage administration, to better mimic the food ingestion and all the molecular, anatomical, histological, and immunological characteristics of food ingestion. Glycemia was measured at the baseline and 1 h after glucose bolus administration with a glucometer (Precision Xtra Meter, Abbott Diabetes Care, Portugal) and test strips (Abbott Diabetes Care, Portugal). In order to determine glucose-specific activity, 20 µL plasma (collected at the baseline and 1 h after glucose administration) was deproteinized with 200 µL ice-cold perchloric acid (0.4 N) and centrifuged, and its radioactivity was measured in a scintillation counter (Tri-Carb 2800TR, Perkin-Elmer, Madrid, Spain). Animals were euthanized 1 h post glucose bolus administration with sodium pentobarbital (60 mg/kg, i. p) and tissues, such as the liver, soleus muscle, and white (WAT: mesenteric, mWAT; epidydimal, eWAT) and brown adipose tissues (BAT), were collected (50–200 mg) and homogenized in 1 ml ice-cold perchloric acid (0.4 N). Tissue samples were centrifuged and 2-deoxy-D-[3H]glucose incorporation was measured in the supernatants by scintillation counting. Tissue glucose uptake (defined as the glucose metabolic index, Rg’) was calculated as described by Sacramento et al. (2017) (Figure 1) (Sacramento et al., 2017).

#### *Ex vivo* tissue-specific glucose uptake

After an episode of overnight fasting, the animals were euthanized with sodium pentobarbital (60 mg/ kg, i. p.) and the liver, soleus, and WAT (mesenteric, mWAT, and epididymal eWAT) (50–150 mg) samples were rapidly collected in an ice-cold 21%O_2_/5%CO_2_-equilibrated tyrode solution (in mM: 140 NaCl, 5 KCl, 2 CaCl2, 1.1 MgCl2, 10 HEPES, and 5.5 glucose, pH 7.40). Three to four pieces of the tissue per Eppendorf tube (2 ml) were then incubated at 37°C for 15 min for stabilization and afterward moved to other tubes for incubation for 15 min per piece in different experimental conditions. Different tissues samples were incubated in the presence and absence of insulin (10 mU ml^−1^) together with dopamine (10 µM); dopamine plus domperidone (50 nM); dopamine plus haloperidol (500 nM), bromocriptine (10 μM, kindly provided by Generis (Portugal)); bromocriptine plus domperidone; and bromocriptine plus haloperidol. The samples were then incubated with 2-deoxy-D-[1,2–3H]-glucose for 30 min and homogenized. Uptake of 2-deoxy-D-[1,2–3H]-glucose was performed as described in the previous section. Results were expressed in cpm.mg tissue^−1^ (Figure 1).

Drug concentrations were chosen with an aim to achieve the complete activation of receptors, and therefore were 10–100x higher than the respective EC50 for agonists and IC50 as described for antagonists. The dopamine concentration tested was based on previous data obtained *in vivo* by our laboratory for the modulation of blood pressure and ventilation Monteiro et al. (2011) and for glucose uptake (Tavares et al., 2020). Bromocriptine was tested in two concentrations that mainly act on D2R (10 and 50 µM) (Beaulieu et al., 2019), but since there were no statistical differences between the doses tested, the dose of 50 µM promoted a less pronounced effect (data not shown), meaning that we have probably reached the maximal efficacy of the drug, or that we are probably producing a desensitization of the receptor, we used at the lowest concentration. Haloperidol was used in a dose that blocks both D1R and D2R (Beaulieu et al., 2019).

### Western Blot for Analysis of IR, Akt, AMPK, HSL, ACC, and ACL Phosphorylation

For Western blot analyses, tissue explants were collected and immediately stored at−80ºC after a 15-min incubation under different experimental conditions. Tissue explants (100 mg/ ml, n = 5–9) were homogenized and processed for Western blot as previously described by Matafome (Matafome et al., 2012). Primary antibodies to the phosphorylated forms of insulin receptor (InsR, Tyr972, ab5678), protein kinase B (PKB), also known as Akt (Akt, Ser473*, #*4058S), adenosine monophosphate kinase (AMPK, Thr172, #2535S), hormone sensitive lipase (HSL, Ser563, #4139S), acetyl CoA carboxylase (ACC, Ser79, #11818P), and ATP citrate lyase (ACL, Ser455 #4331P) were obtained from Abcam (Cambridge, United Kingdom) or Cell Signalling Technology (Danvers, United States). Calnexin (AB0041-200) from SICGEN (Cantanhede, Portugal) was used as the loading control. Secondary antibodies of anti-rabbit and anti-goat samples were obtained from Bio-Rad (Spain) and Invitrogen or Thermo Fisher Scientific (United States), respectively. Membranes were revealed using an ECL substrate (Advansta, CA, United States) in a VersaDoc system (Bio-Rad, United States) and analyzed with ImageQuant® (Molecular Dynamics, United States).

### Data Analysis and Statistical Procedures

In glucose uptake experiments, a piece of each evaluated tissue (from a minimum of five animals per condition) was used and incubated with dopamine or its agonists and antagonists. For statistical analyses, all data were evaluated using GraphPad Prism Software, version 8 (GraphPad Software Inc., United States) and presented as scatter plots or mean values with standard deviation (SD). Significance between the mean values was calculated by the one-way ANOVA with Dunnets’s and Bonferroni multiple comparison tests. Differences were considered significant at *p* < 0.05.

## Results

**FIGURE 2:**
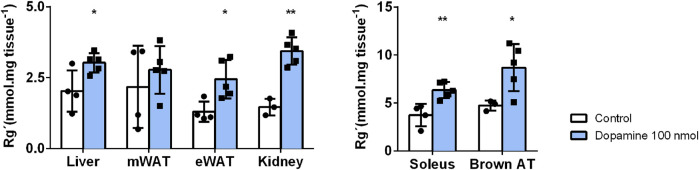
Effects of *in vivo* dopamine administration on glucose uptake in insulin-sensitive tissues. Exogenous dopamine administration at 100 nmol enhances glucose uptake significantly in all tissues except for mWAT. Values and bars represent mean ± SD. One-way ANOVA with Dunnet’s post hoc comparisons test was performed, where * indicates different from the control group. Levels of significance: *p < 0.05; **p < 0.01; ***p < 0.001. mWAT, mesenteric white adipose tissue; eWAT, epididymal white adipose tissue; and BAT, brown adipose tissue.

Peripheral dopamine mediates *in vivo* tissue glucose uptake. Dopamine (100 nmol) was administered intravenously immediately before oral glucose administration. Figure 2 presents the effect of dopamine on glucose uptake in insulin-sensitive tissues. The soleus muscle and BAT exhibited a higher glucose uptake in relation to the rest of the tissues, showing that these are tissues with a high rate of glucose uptake (baseline liver = 2.03; mWAT = 2.18; eWAT = 1.30; Soleus = 3.73; and BAT = 4.73 mmol/ mg tissue). Dopamine significantly increases glucose uptake in all the tissues tested, except mWAT, producing an increase of 49% in the liver, 88% in eWAT, 70% in the soleus muscle, and 88% in BAT.

### Dopamine Directly Acts in Insulin-Sensitive Tissues Through Different Receptors to Regulate Glucose Uptake

Aiming to clarify the contribution of the specific dopamine receptors role in each insulin-sensitive tissue, ex *vivo* tissue uptake of glucose studies was performed; thus, insulin-sensitive tissues were incubated with selective dopamine receptor’s agonists and antagonists.

**FIGURE 3:**
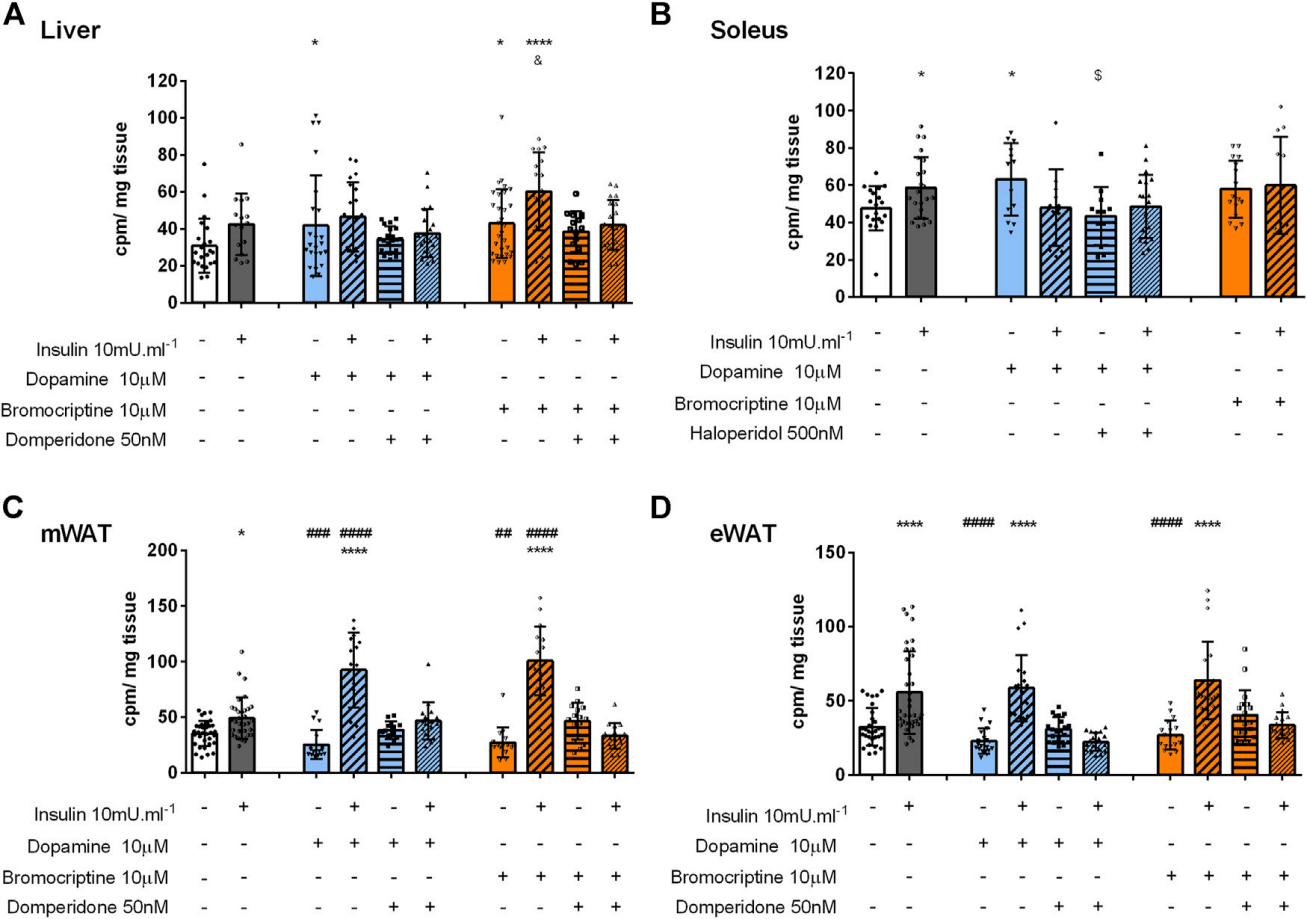
Role of dopamine and bromocriptine on *ex vivo* glucose uptake in the liver **(A)**, soleus muscle **(B)**, mWAT **(C),** and eWAT **(D)**. The effect of dopamine (10 μM, blue bars) and bromocriptine, a D2R agonist (10 μM, orange bars), on 2-deoxy-D-[1,2–3H]-glucose uptake in the presence and absence of insulin (10m U ml-1) and its blockade by domperidone, a D2R antagonist (50 nM) and/or haloperidol (500 nM) a D1R and D2R antagonist in the liver, the soleus muscle, mWAT, and eWAT. Bars represent means ± SD. One-way ANOVA with Dunnet’s post hoc comparison test * *vs.* control; # *vs.* insulin; $ *vs.* dopamine, & *vs.* bromocriptine. Levels of significance: * *p* < 0.05; **p < 0.01; ***p < 0.001; **** *p* < 0.0001. mWAT, mesenteric white adipose tissue; eWAT, epididymal white adipose tissue; D1R, dopamine receptor 1; and D2R, dopamine receptor type 2.

Dopamine effect on glucose uptake in the liver: Insulin significantly increased glucose uptake by 37% in the liver (control = 31 cpm min^−1^) (Figure 3A). Dopamine alone or in the presence of domperidone did not change glucose uptake in the liver (Figure 3A). However, dopamine + insulin-induced increase of glucose uptake was abolished by domperidone (26% decrease, Figure 3A), suggesting that dopamine D2R has a role in the insulin effect on glucose uptake in the liver. This was further suggested by the significant increase in glucose uptake by 48% in response to bromocriptine, an effect that was independent and additive to insulin-induced glucose uptake (Figure 3A). Domperidone inhibited this effect of bromocriptine, confirming the dopamine D2R-mediated effect.

Dopamine induces glucose uptake in the skeletal muscle *via* dopamine D1R: The skeletal muscle is a major organ involved in glucose homeostasis and therefore herein we evaluated the role of dopamine signaling in skeletal muscle glucose uptake. Similar to insulin, dopamine applied alone significantly increased glucose uptake by 47%, an effect not mimicked by bromocriptine stimulation, suggesting a dopamine D1R-mediated mechanism (Figure 3B). This was confirmed using haloperidol, in a dose that blocks both dopamine D1 and D2 receptors, as it inhibits the effect of dopamine on glucose uptake.

Dopamine potentiated insulin-induced glucose uptake in mWAT *via* dopamine *D2R*: Finally, to understand the role of dopaminergic signaling in WAT depots with different metabolic functions, mWAT and eWAT were also studied ex *vivo*. Dopamine or bromocriptine did not alter glucose uptake in both tissues (Figures 3C,D). Nevertheless, in mWAT but not in eWAT, both dopamine and bromocriptine potentiated insulin-mediated glucose uptake (38 and 72% *vs*. 162 and 185%, respectively) in comparison with the control group (35 cpm/ mg tissue, Figure 3C). This last effect was blocked by the presence of domperidone, confirming a dopamine D2R-dependent mechanism. Interestingly dopamine and bromocriptine did not change the ex *vivo* glucose uptake or potentiate the effect of insulin (Supplemental figure 1A).

### Dopamine Differently Regulates Insulin Signaling in Insulin-Sensitive Tissues

**FIGURE 4:**
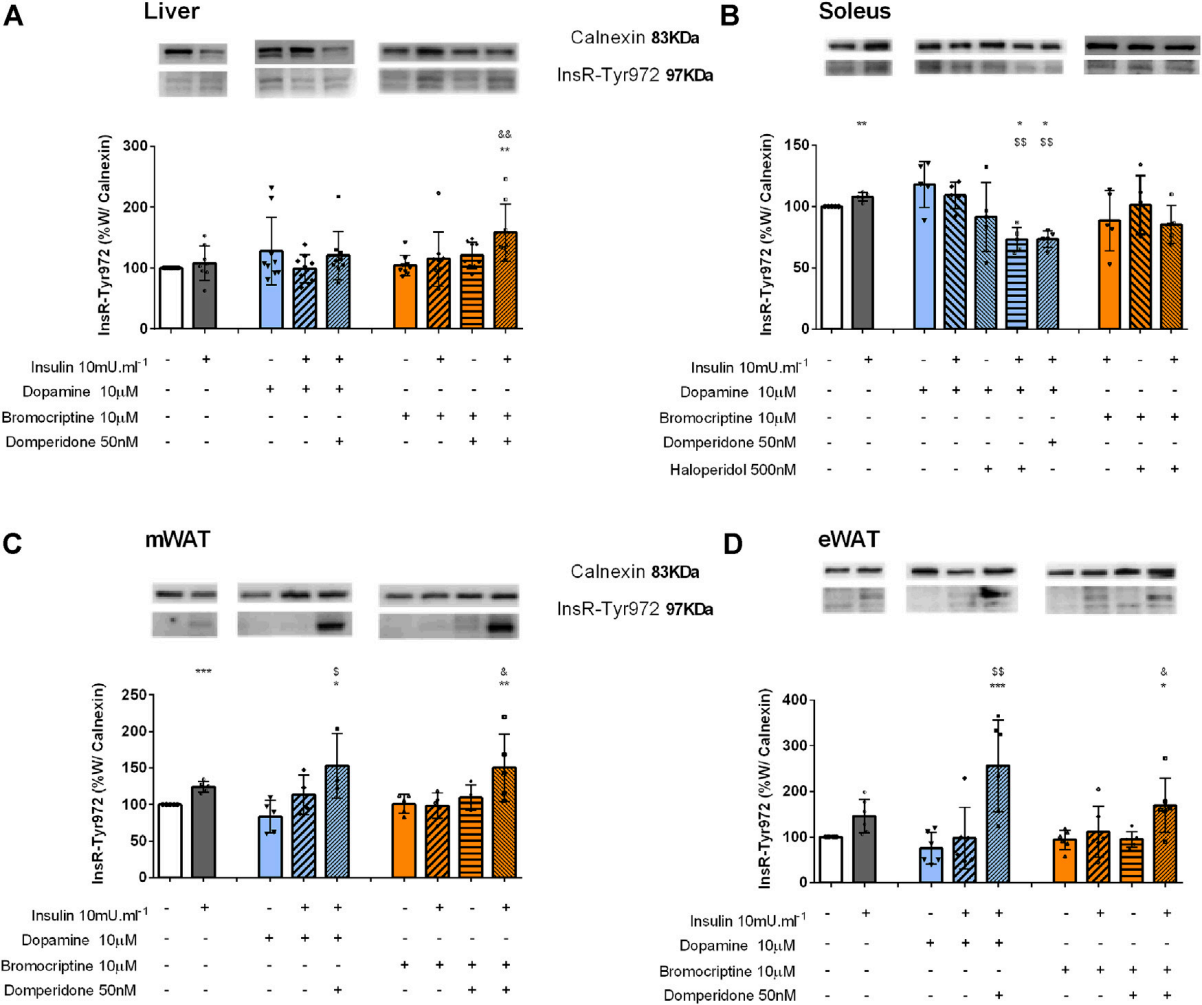
Effect of dopamine and bromocriptine on insulin receptor phosphorylation in the liver **(A)**, soleus muscle **(B)**, mWAT **(C)**, and eWAT **(D)**. Effect of dopamine (10 μM, blue bars) and bromocriptine, a D2R agonist (10 μM, orange bars), on insulin receptor phosphorylation (InsR) in the presence and absence of insulin (10m U ml^−1^) and its blockade by domperidone, a D2R antagonist (50 nM) and/or haloperidol (500 nM), a D1R and D2R antagonist in the liver, the soleus muscle, mWAT, and eWAT. The **top** of the figures show representative Western blots for the effect of dopamine and bromocriptine on InsR-Tyr972 (97 kDa band) phosphorylation, in the presence and absence of insulin and its blockade by domperidone and/or haloperidol. Calnexin was used as the loading control (83 kDa band). Bars represent mean ± SD of the expression of InsR-Tyr972. On the **bottom** of the bars are shown the different conditions used for each group tested with + meaning presence of that stimuli and – absence of it. One-way ANOVA with Dunnet’s post hoc comparison test * vs control; # *vs*. insulin; $ *vs*. dopamine; & *vs*. bromocriptine. Levels of significance: * *p* < 0.05; **p < 0.01; ***p < 0.001; **** *p* < 0.0001. mWAT, mesenteric white adipose tissue; eWAT, epididymal white adipose tissue; D1R, dopamine receptor type 1; and D2R, dopamine receptor type 2.

Bromocriptine-induced glucose uptake in the liver is independent of the InsR. To disclose the role of dopamine in insulin signaling, the levels of InsR phosphorylated at Tyr972 (InsR-Tyr972) were determined by Western blot. As depicted in Figure 4A, incubation with dopamine and bromocriptine did not change liver InsR-Tyr972 levels. However, since domperidone in the presence of bromocriptine and insulin increased the phosphorylation of InsR by 58% in comparison with the control group and 51% in comparison with bromocriptine (Figure 4A), we can speculate that dopamine D2R is involved in other InsR-induced mechanisms different from glucose uptake regulation.

Dopamine induces InsR phosphorylation in the skeletal muscle: In the skeletal muscle, insulin and dopamine applied separately were able to induce InsR phosphorylation, by 8 and 18%, but when applied together the effect was not modified, suggesting that they act on the same signaling pathway (Figure 4B). Haloperidol decreased dopamine and dopamine plus insulin effects on InsR-Tyr972 levels by 22 and 38%, respectively (Figure 4B), an effect mimicked by domperidone, a selective dopamine D2R agonist (Figure 4B).

Dopamine-induced potentiation of insulin action in WAT is independent of the InsR*:* Insulin significantly increased InsR phosphorylation by 24% in the mWAT and by 46% in eWAT, respectively (Figures 4C,D), but not in BAT (Supplemental figure 1B). Dopamine or bromocriptine alone or in the presence of insulin did not alter InsR-Tyr972 phosphorylation in both tissues (Figures 4C,D). Nevertheless, an increase in the InsR-Tyr972 levels in mWAT and eWAT was observed when domperidone was added to the condition dopamine + insulin (82 and 274%) and bromocriptine + insulin (49 and 81%) (Figures 4C,D), suggesting that, as observed in the liver, dopamine D2R plays a role in other InsR-induced mechanisms than glucose uptake.

### Dopamine Regulates AMPK Activation in Insulin-Sensitive Tissues

**FIGURE 5:**
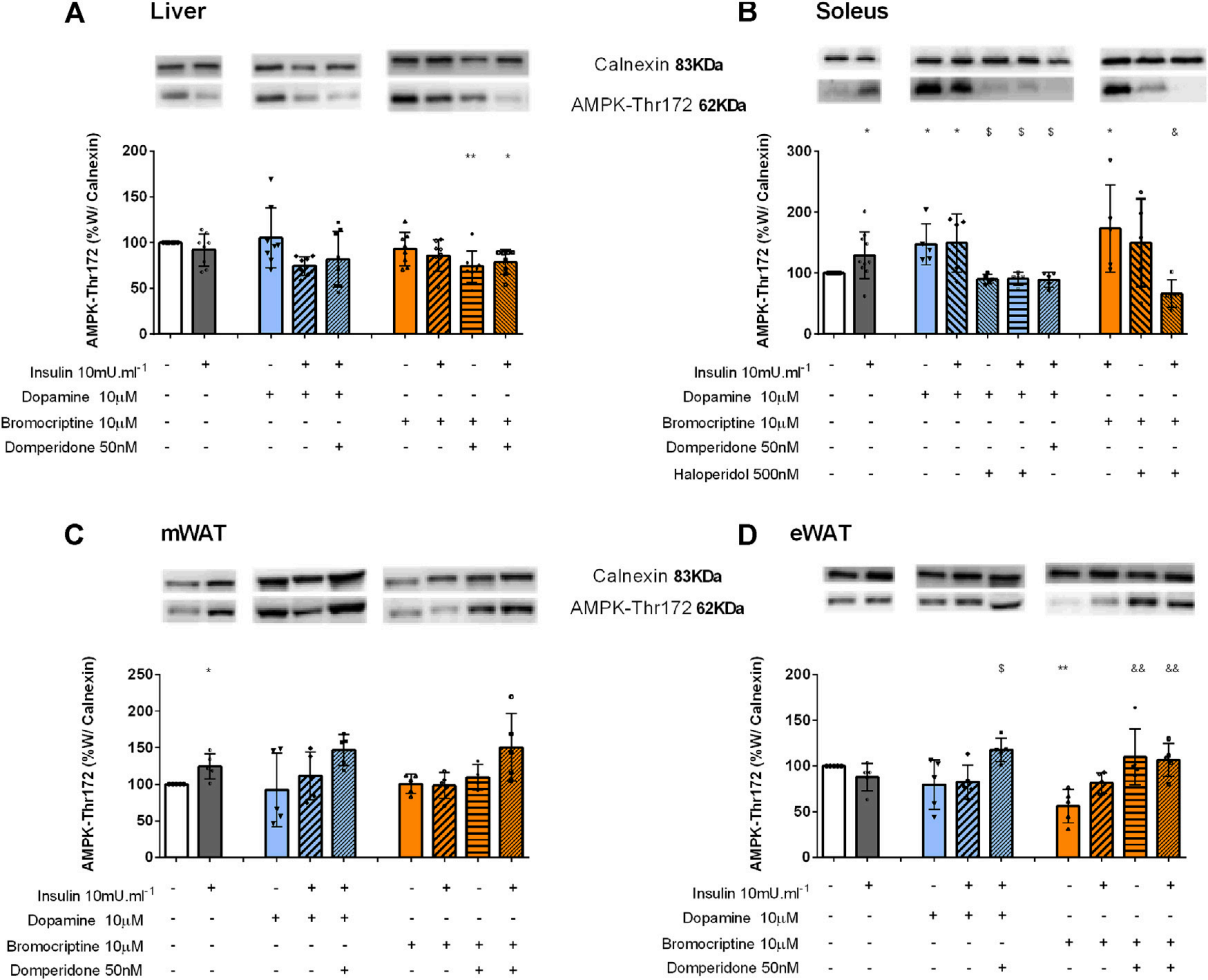
Involvement of dopamine and bromocriptine on AMPK phosphorylation in the liver **(A)**, soleus muscle **(B)** mWAT **(C),** and eWAT **(D)**. Effect of dopamine (10 μM, blue bars) and bromocriptine (10 μM, orange bars) on AMPK phosphorylation in the presence and absence of insulin (10m U ml^−1^) and its blockade by domperidone, a D2R antagonist (50 nM) and/or haloperidol (500 nM), a D1R and D2R antagonist in the liver, soleus muscle, and mesenteric and epididymal adipose tissue. The** top** of figures shows representative Western blots for the effect of dopamine and bromocriptine on AMPK-Thr172 (62 kDa band) phosphorylation, in the presence and absence of insulin, and its blockade by domperidone and/or haloperidol. Calnexin was used as the loading control (83 kDa band). Bars represent mean ± SD. On the **bottom** of the bars are shown the different conditions used for each group tested with + meaning presence of that stimuli and – absence of it. One-way ANOVA with Dunnet’s post hoc comparison test * *vs*. control; # *vs*. insulin; $ *vs*. dopamine; & *vs*. bromocriptine. Levels of significance: * *p* < 0.05; **p < 0.01; ***p < 0.001; **** *p* < 0.0001. mWAT, mesenteric white adipose tissue; eWAT, epididymal white adipose tissue; D1R, dopamine receptor type 1; and D2R, dopamine receptor type 2.

Dopamine D2R is involved in the AMPK function in the liver: We also evaluated the impact of dopaminergic signaling on AMPK activation, an energy sensor that has a key role in the control of energy metabolism, by regulating hepatic glucose and lipid metabolism, through Thr172 phosphorylation. In the liver, a significant reduction of 26 and 21% was observed when domperidone was added to bromocriptine and to bromocriptine + insulin, respectively, in comparison with the control group. This suggests that dopamine D2R activation is necessary for AMPK function in the liver (Figure 5A).

Dopamine activates AMPK in the skeletal muscle*:* Insulin significantly increased AMPK phosphorylation by 29% in the soleus muscle (Figure 5B). Dopamine and bromocriptine alone increased by 47 and 73% AMPK phosphorylation levels, effects that were not modified in the presence of insulin (Figure 5B). These effects were blocked by haloperidol and domperidone–dopamine D1R + D2R and selective dopamine D2R antagonists, respectively, suggesting that the effects of dopamine and bromocriptine on AMPK phosphorylation are mediated by dopamine D2 receptors.

Dopamine D2R inhibits AMPK activation in the adipose tissue: In mWAT, dopamine and bromocriptine did not alter AMPK-Thr172 levels compared to the control group (Figure 5C). On the other hand, in eWAT, while dopamine did not affect AMPK-Thr172 phosphorylation, bromocriptine decreased AMPK-Thr172 phosphorylation by 44% when compared to the control group (Figure 5D). Incubation with dopamine or bromocriptine in the presence of domperidone + insulin significantly increased the levels of AMPK-Thr172 in eWAT by 48 and 90%, respectively (Figure 5D), and a similar trend was observed in mWAT (Figure 5C). Such results suggest an inhibitory effect of dopamine D2R on AMPK phosphorylation on the white adipose tissue. No significant changes were observed for the effects of dopamine and/or bromocriptine on AMPK phosphorylation in BAT (Supplemental figure 1C).

**FIGURE 6:**
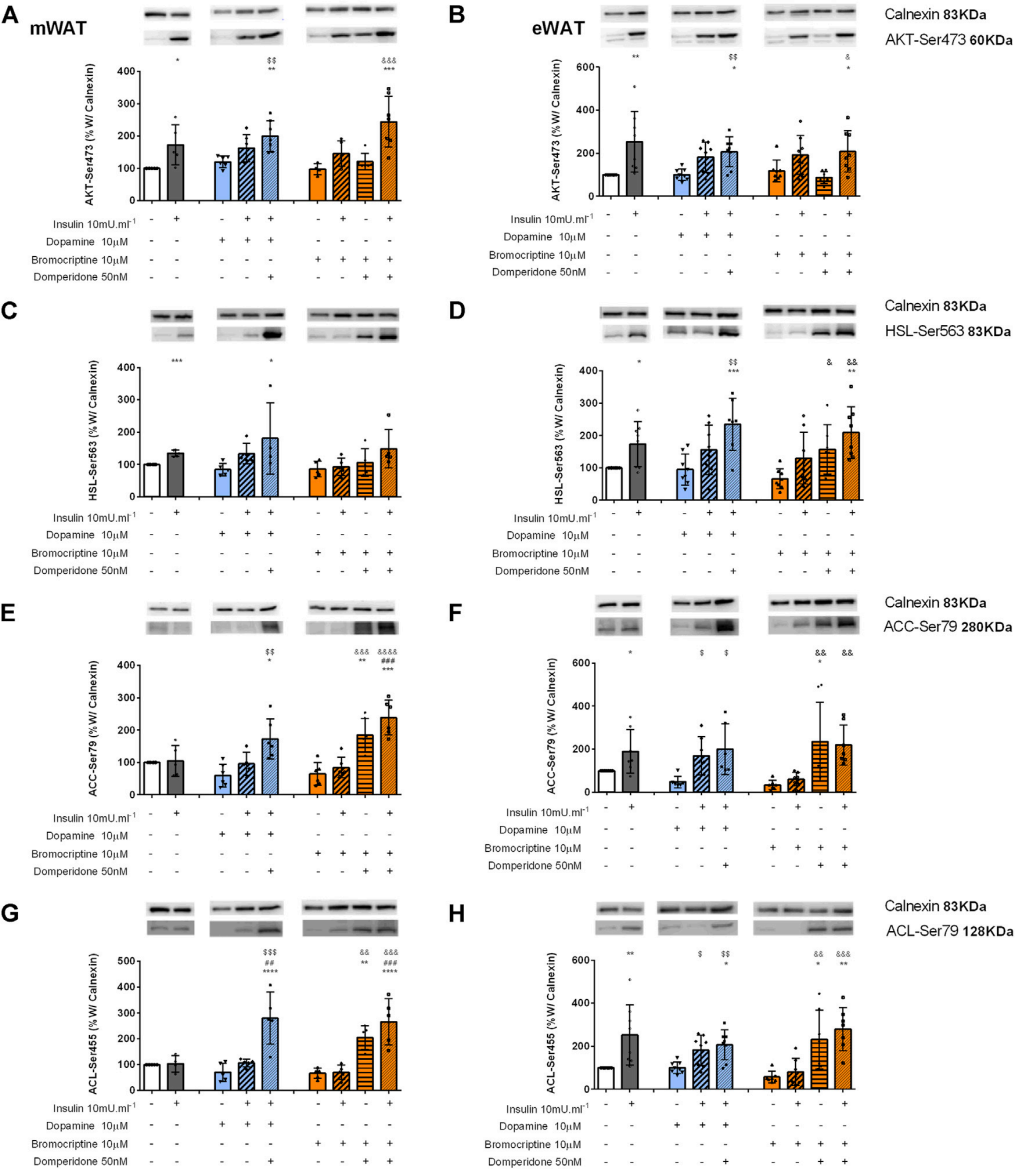
Involvement of dopamine and bromocriptine on Akt **(A,B)**, HSL **(C,D)**, ACC **(E,F)**, and ACL **(G,H)** phosphorylation in mWAT and eWAT, respectively. Figures show the effect of dopamine (10 μM, blue bars) and the effect of bromocriptine (10 μM, orange bars) on mWAT and eWAT. Akt **(A,B)**, HSL **(C,D)**, ACC **(E,F)**, and ACL **(G,H)** phosphorylation, and its blockade by domperidone (50 nM). The insulin dose used was 10m U ml^−1^. The **top** of figures show representative Western blots for the effect of insulin, dopamine, and bromocriptine in the presence or absence of domperidone, a D2R antagonist, on Akt-Ser743 **(A,B)**, HSL-Ser563 **(C,D)**, ACC-Ser79 **(E,F)**, and ACL-Ser455 **(G,H)** phosphorylation, where the bands on the top represent calnexin (83 kDa band), the loading control, and the bands below show the proteins of interest–Akt-Ser743, HSL-Ser563, ACC-Ser79, and ACL-Ser455. Bars represent means ± SD. On the **bottom** of the bars are shown the different conditions used for each group tested with + meaning presence of that stimuli and – absence of it. One-way ANOVA with Dunnet’s post hoc comparison test * *vs*. control; # *vs*. insulin; $ *vs*. dopamine; & *vs*. bromocriptine. Levels of significance: * *p* < 0.05; **p < 0.01; ***p < 0.001; **** *p* < 0.0001. mWAT, mesenteric white adipose tissue; eWAT, epididymal white adipose tissue; and D2R, dopamine receptor type 2.

Inhibition of dopamine D2 receptor increases dopamine-mediated stimulation of adipose tissue lipid catabolic pathway**s**. As expected, Akt-Ser473 phosphorylation increased with insulin incubation in both mWAT and eWAT (73 and 152%, respectively) compared to control conditions (Figures 6A,B, respectively). Dopamine or the dopamine D2R agonist, bromocriptine when applied alone did not increase Akt-Ser473 phosphorylation levels in both WAT depots or changed insulin-mediated increase in Akt-Ser473 levels (Figures 6A,B). These results suggest that both dopamine and bromocriptine exert their actions independent of insulin receptor pathway activation, since Akt-Ser473 is a downstream modulator of InsR signaling (Figures 6A,B).

To understand the implications of dopaminergic signaling on lipid metabolism pathways, we evaluated HSL, ACC, and ACL activation through the measurement of their phosphorylated forms: HSL-Ser563, ACC-Ser79 (inhibitory), and ACL-Ser455. Insulin increased by 35% and HSL-Ser563 levels by 73% in mWAT and eWAT depots, respectively (Figures 6C,D). Interestingly, in both fat depots, dopamine or bromocriptine alone did not change HSL-Ser563 levels Figures 6C,D), but in the presence of domperidone + insulin, HSL-Ser563 levels significantly increased in mWAT and eWAT compared to dopamine (114 and 148%) or to bromocriptine (73%, and 217%), respectively (Figures 6C,D).

Interestingly, both dopamine and bromocriptine directly decreased by 50 and 66%, respectively, ACC-Ser79 phosphorylation levels in eWAT, meaning that these substances decreased ACC activity (Figures 6E,F). This effect was not observed in mWAT (Figures 6E,F). ACC-Ser79 levels were significantly increased in mWAT and eWAT in the presence of domperidone in combination with all the other tested conditions (Figures 6E,F), which is consistent with increased AMPK activation in the same condition, a known inhibitor of ACC. Dopamine and bromocriptine did not present a direct effect on ACL phosphorylation, but domperidone in the presence of insulin significantly increased ACL-Ser455 levels in mWAT and eWAT by 298 and 156%, respectively, when compared to dopamine, and by 304 and 390%, respectively, when compared to bromocriptine (Figures 6G,H).

Altogether, our data suggest that dopamine D2R is involved in glucose uptake–independent activation of InsR and in the regulation of pathways associated with lipid metabolism in WAT, by impacting on the expression of phosphorylated forms of AMPK, HSL, ACC, and ACL in both WAT depots.

## Discussion

Herein, we described for the first time the direct effects of dopamine (mediated by different dopamine receptor activation) in glucose uptake and metabolism in insulin sensitivity tissues. We showed that peripheral dopamine stimulates glucose uptake in several tissues, in part through direct actions in the liver, the skeletal muscle, and WAT. Moreover, dopamine receptors, especially dopamine D2R, play a role in these tissues in the regulation of InsR, Akt, and AMPK phosphorylation. Also, dopamine D2R is involved in the regulation of lipid metabolism pathways in WAT, which could be relevant for unraveling new therapeutic approaches for obesity-related diseases. Herein, we also show that dopamine effects on glucose uptake occur preferentially through dopamine D1R in the skeletal muscle and through dopamine D2R in the liver and WAT (Table 1).

**TABLE 1:** Summary of the findings supporting the involvement of peripheral dopamine and its receptors on glucose uptake, insulin action, AMPK signaling, and lipid storage. Peripheral dopamine stimulates glucose uptake in the liver, the skeletal muscle, and white adipose tissue (WAT). Dopamine directly acts in the same tissues to regulate InsR, Akt, and AMPK phosphorylation, being also involved in the regulation of metabolic functions in WAT, which can be an important factor for obesity-related diseases. Dopamine-directed effects in insulin-sensitive tissues are differentially modulated by D1 and D2 dopamine receptors.

Tissue	Parameter	Dopamine direct effect	D1 effect	D2 effect
mWAT	Glucose uptake	**=**	**=**	**↑**
	Insulin signaling			**=**
	AMPK signaling			**=**
	Lipid storage			**↑**
eWAT	Glucose uptake	**=**	**=**	**=**
	Insulin signaling			**=**
	AMPK signaling			**↓**
	Lipid storage			**↑**
Liver	Glucose uptake	**=**	**=**	**↑↑**
	Insulin signaling	**=**		**=**
	AMPK signaling	**↑**		**↑**
Skeletal muscle	Glucose uptake	**↑**	**↑↑**	**=**
	Insulin signaling	**↑**	**=**	**↑**
	AMPK signaling	**↑**	**=**	**↑**

Dopamine effects on glucose metabolism were attributed for many years to the regulation of the central nervous system (DeFronzo, 2011; Lopez Vicchi et al., 2016), supported by the alterations in peripheral metabolism produced by antipsychotics (Lopez Vicchi et al., 2016; Freyberg and McCarthy, 2017). In agreement, the effects of bromocriptine, a dopamine D2 receptor agonist, on glucose metabolism have been attributed to the central regulation of sympathetic nervous system activity (DeFronzo, 2011). However, with the identification of dopamine receptors in the pancreas and in the adipose tissue, and the description of its involvement in the regulation of insulin and adipokines secretion (Borcherding et al., 2011; Chaudhry et al., 2016; Farino et al., 2019), the peripheral role of dopamine needs to be considered. The results obtained in the present study agree with the role of peripheral dopamine on glucose metabolism, as we show a direct effect of dopamine on tissues outside the central nervous system in the regulation of glucose and lipid metabolic pathways. Moreover, both dopamine receptors participate in this modulation playing distinct roles in the different insulin-sensitive tissues.

We showed *in vivo* that peripheral dopamine administration during an OGTT produces an overall enhancement of tissue glucose uptake by the insulin-sensitive tissues in the liver, WAT (mesenteric and epididymal), the soleus muscle, and BAT suggesting that dopamine is involved in the peripheral glucose homeostasis. We also found that dopamine directly promotes peripheral glucose uptake in insulin sensitive tissues ex *vivo*, *via* its action on different dopamine receptor subtypes. Interestingly, increased *in vivo* glucose uptake in each tissue after dopamine administration does not resemble the ex *vivo* glucose uptake in the same tissues upon direct dopamine or bromocriptine stimulation, showing that dopamine may have direct and indirect effects on each of these tissues. For example, we can postulate that when administered systemically *in vivo*, dopamine may act to decrease sympathetic activation to efferent organs changing the metabolic turnover and the release of signaling molecules that will act distant from the releasing cells, as seen previously with other mediators (Guilherme et al., 2019). An example of this kind of mechanism is the regulation of adipokines secreted by WAT that will act on BAT to change its metabolic status (Guilherme et al., 2019). Another counter-regulatory mechanism that we can postulate to be involved in the regulation of peripheral dopamine effects could be the modulation of parasympathetic activity through the vagus nerve. It is known that the parasympathetic vagus nerve mediates changes in BAT sympathetic activity and energy expenditure in response to altered efferent signaling coming from tissues such as the liver and WAT (Madden and Morrison, 2016). Finally, we can speculate that the use of alternative fuels by the other tissues in *in vivo* experiments can be responsible for the forwarding of glucose to BAT and consequent increase in glucose uptake (Stanley et al., 2019).

Herein, we also found for the first time differences on the dopamine receptors involved in the regulation of glucose uptake measured ex *vivo*. The dopamine D2R agonist directly stimulated glucose uptake in the liver, while in the soleus, even though dopamine stimulates glucose uptake, we cannot find any alterations with bromocriptine incubation, pointing out an involvement of dopamine D1 receptors in glucose uptake in the skeletal muscle. In the adipose tissue, dopamine D2R stimulation only potentiated the effect of insulin on glucose uptake, independently of InsR activation. Our results suggest an important and tissue-specific role of dopamine in the regulation of glucose uptake. Moreover, our results raised some questions or hypotheses regarding the involvement of dopamine receptors in the regulation of glucose uptake and glucose transporters, GLUTs. Given the difference between glucose transporters in the liver (insulin-independent GLUT2) and the muscle and adipose tissue (insulin-dependent GLUT4) (Navale and Paranjape, 2016), it seems expectable that dopamine-mediated glucose uptake may be dependent on the type of GLUT translocated to the plasma membrane.

Interestingly, domperidone induced InsR-Tyr972 phosphorylation in the liver and InsR-Tyr972 and Akt-Ser473 phosphorylation in WAT without any evident increase in glucose uptake, suggesting that the dopamine D2R impact on insulin signaling on these tissues exerts other metabolic effects in cells apart from glucose uptake. Altogether, the results of dopamine receptors modulation herein described point toward a major role of dopamine D2R in the peripheral control of glucose metabolism in the insulin-sensitive tissues with the exception of the soleus muscle where dopamine D1R appears to be a modulator of glucose uptake.

Additionally, from our results, it is clear that dopamine D2R potentiated insulin–glucose uptake at mWAT, suggesting an interaction between insulin and dopamine signaling pathways to control glucose uptake in this tissue, while in the soleus muscle and the liver the effect seems to be additive. This is corroborated by the fact that dopamine- and bromocriptine-mediated glucose uptake in WAT is independent of InsR and Akt activation (downstream regulator of InsR pathway signaling) since phosphorylation of both proteins is only stimulated by insulin. Dopamine is known to act on dopamine D1R and D2R to regulate cAMP levels inside the cell, regulating neuronal excitability through membrane availability of ion channels (Savica and Benarroch, 2014). Apart from the classical activation of the Gαi/o protein coupled to dopamine D2R and thereby the inhibition of adenylate cyclase, it is known that dopamine D2R has several effectors (Thummel, 2005) that can account for the effects herein observed for dopamine in WAT.

In the present study, we also describe for the first time that dopamine receptors regulate AMPK phosphorylation in eWAT and in the skeletal muscle. Interestingly, these effects were opposite since dopamine decreased AMPK phosphorylation in eWAT *via* dopamine D2R, while enhanced AMPK phosphorylation in the soleus muscle upon stimulation with dopamine and bromocriptine. Confirming the dopamine D2R modulation, the effects of dopamine D2R agonism on the soleus muscle AMPK phosphorylation were reversed by D2R antagonism.

We also show that dopamine D1 receptors modulate glucose uptake in the skeletal muscle, suggesting different pathways mediating metabolism in the soleus muscle. Nevertheless, we cannot forget that AMPK also facilitates GLUT4 translocation to the plasma membrane (Wolfe, 1998; Mihaylova and Shaw, 2011) and therefore we can speculate that D2R modulation might contribute indirectly to glucose uptake as well. The exact mechanism by which dopamine D2R increases AMPK-Thr172 levels in the skeletal muscle is not clearly understood and therefore more experiments will be needed in the future to clarify this.

On the other hand, dopamine D2R inhibition by domperidone in the presence of dopamine increased HSL, ACC, and ACL phosphorylation, and therefore activates HSL and ACL pathways and inactivates the ACC pathway, in both mWAT and eWAT showing dopamine involvement in lipid metabolism pathway modulation in the adipose tissue. HSL and ACL are involved in lipid catabolism pathways, so we can speculate that dopamine D2R inhibition promotes lipid oxidation in the adipose tissue. Regarding ACC, its main function is to catalyze lipogenesis, and therefore domperidone, by inactivating this enzyme, will promote a decrease in fatty acid synthesis and a shift of lipid metabolism in lipolytic pathways. This, together with HSL and ACL activation, will improve lipid metabolism in the adipose tissue and therefore will benefit metabolic disease treatment. Moreover, we recently described that bromocriptine treatment in GK rats, a type 2 diabetes rat model, upregulates catabolic pathways, improving the metabolic profile (Tavares et al., 2021). Altogether, we can clearly say that dopamine receptors modulate lipid metabolism in the adipose tissue, although one might find these results contradictory. However, we must take into account that in the present study, we are evaluating the acute effects of dopamine and the modulation of its receptors; moreover, we are in the absence of metabolic disease, which is described to alter dopamine receptors expression in the adipose tissue (Tavares et al., 2021).

Of note, we cannot forget the possibility of dopamine receptor heterodimerization on the interpretation of the results obtained in the present study, particularly between D1R and D2R. The formation of heterodimers between D2R and D1R are well established in the brain as described, for instance, by Abraham et al. (2014); however, there are no studies regarding this type of heterodimerization in the peripheral organs. Yet, an interaction between those on the activation of adenylyl cyclase and on the production of cAMP has been postulated to regulate insulin secretion from beta cells Bucolo et al. (2019) and to be on the basis of some of the bromocriptine effects in the periphery (Bucolo et al., 2019). Also, interestingly, heterodimerization between D2–D3 receptors has been found in the pancreas. These receptors belong to the same subtype family—the D2-like family—enabling the intensification/enhancement of the intracellular pathway response, in contrast to what happens with a D2R–D1R heterodimerization (Abraham et al., 2014; Maffei et al., 2015; Farino et al., 2019). Moreover, we cannot also forget that dopamine D2R receptors are known to heterodimerize with adenosine and serotonin receptors (Conde et al., 2008; Łukasiewicz et al., 2010; Feltmann et al., 2018; Bucolo et al., 2019) and that adenosine is known to play a major role in glucose homeostasis (Sacramento et al., 2020).

Overall, we found that peripheral dopamine modulates glucose uptake through direct and indirect actions in insulin-sensitive tissues. Dopamine D1R and D2R are differentially involved in glucose uptake and regulation of the insulin signaling pathway and AMPK phosphorylation in insulin-sensitive tissues. Particularly, dopamine *via* D1R regulates glucose uptake in the soleus muscle, while *via* D2R regulates glucose uptake in the liver and WAT. Importantly, we found that dopamine also impacts lipid metabolism pathways in WAT, showing its importance for obesity. Nevertheless, the direct effects of dopamine only partially explain the tissue glucose uptake observed after its *in vivo* peripheral administration. Given that dopamine is not able to cross the blood–brain barrier, other peripheral indirect mechanisms of glucose homeostasis regulation by dopamine may be involved and should be addressed in the future. The present study adds knowledge about the peripheral dopaminergic signaling role over glucose uptake to the already described mechanisms. These results can support the effects of bromocriptine treatment in type 2 diabetes and contribute to the identification of new therapeutic strategies, based on the modulation of dopaminergic signaling, for the treatment of metabolic disorders such as type 2 diabetes, obesity, and metabolic syndrome.

## Data Availability

The original contributions presented in the study are included in the article/Supplementary Material. Further inquiries can be directed to the corresponding author.
