# Supplementary material for: Peripheral Dopamine Directly Acts on Insulin-Sensitive Tissues to Regulate Insulin Signaling and Metabolic Function
Source: Front Pharmacol. 2021 Sep 9;12:713418. doi: 10.3389/fphar.2021.713418 (PMC8458637; doi:10.3389/fphar.2021.713418)

Figure 3

W.B. original membranes

A) Liver

InsR-Tyr972

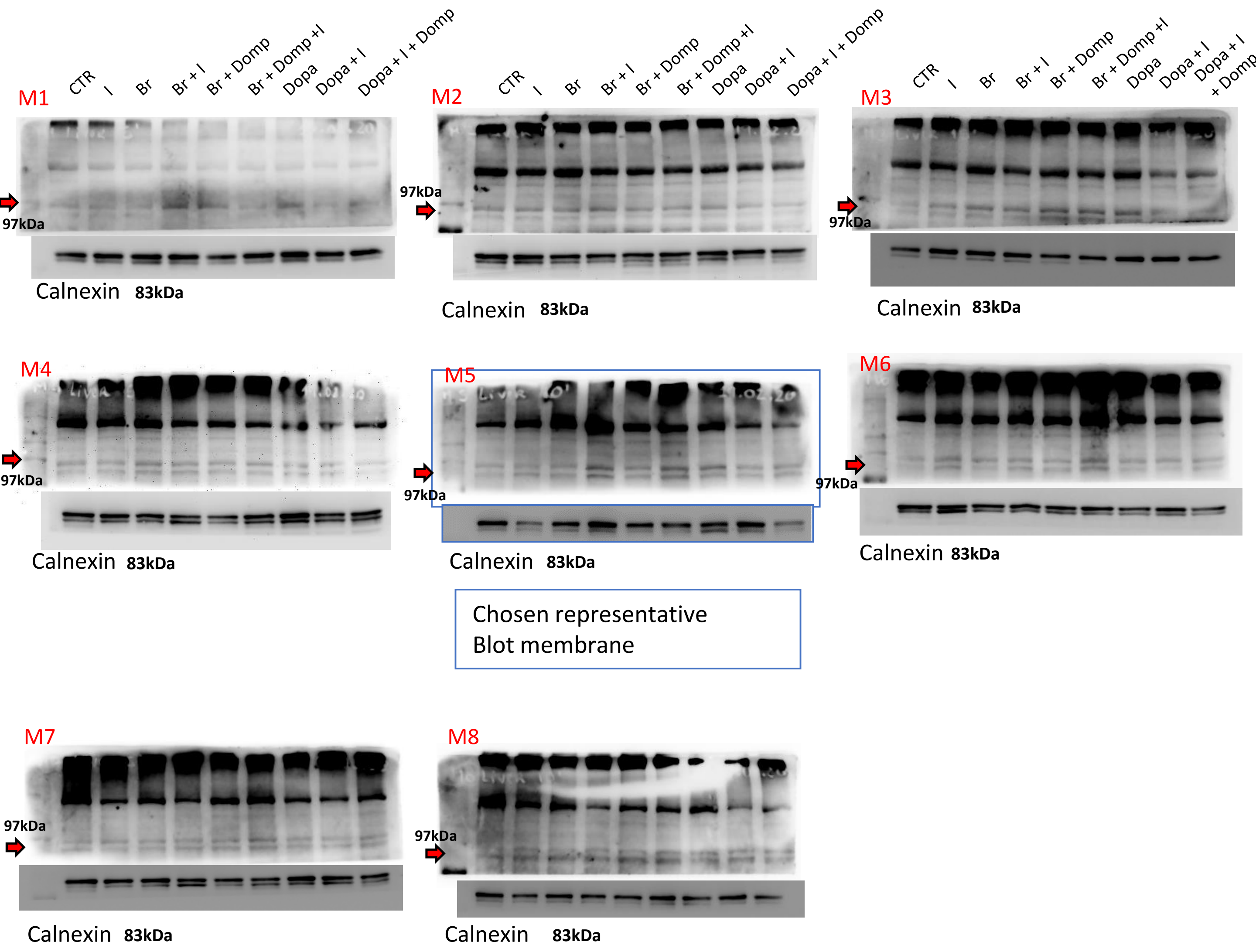

Figure 3

W.B. original membranes

B) Soleus muscle

InsR-Tyr972

Dopamine graphs

M4

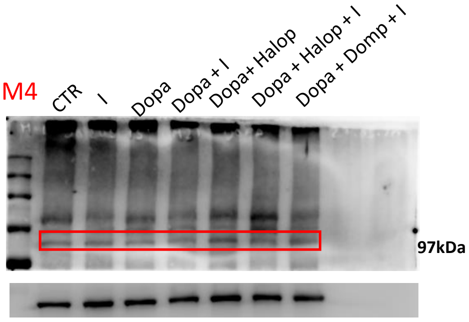

M5

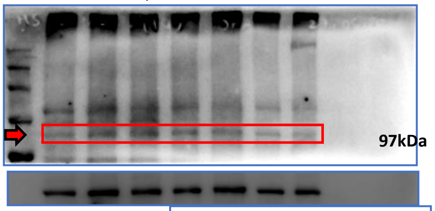

Chosen representative Blot membrane for Dopa

M6

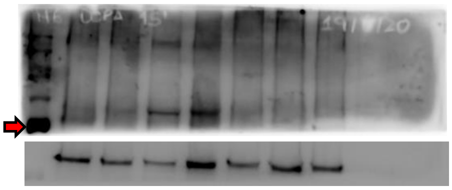

M7

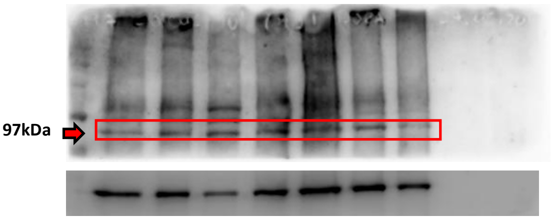

M8

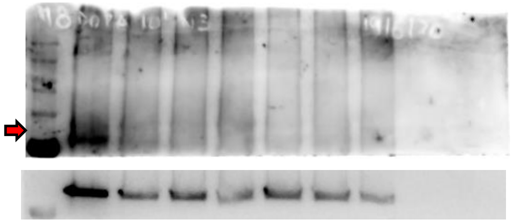

Bromocritine graphs

M1

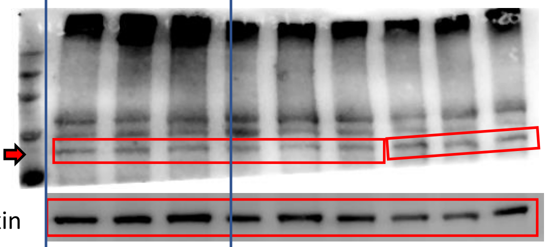

Chosen representative Blot membrane for Dopa

M2

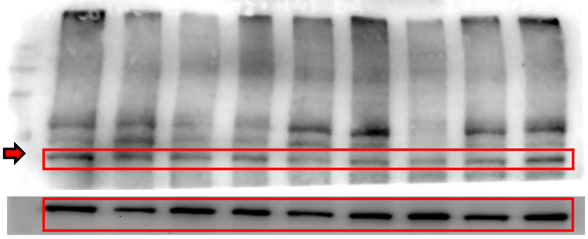

M3

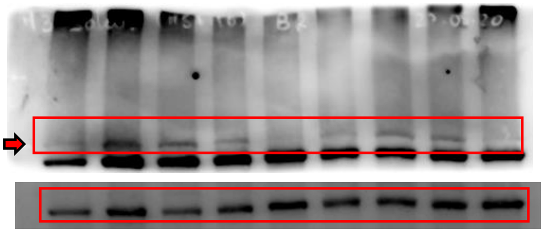

Figure 3

W.B. original membranes

C) mWAT

InsR-Tyr972

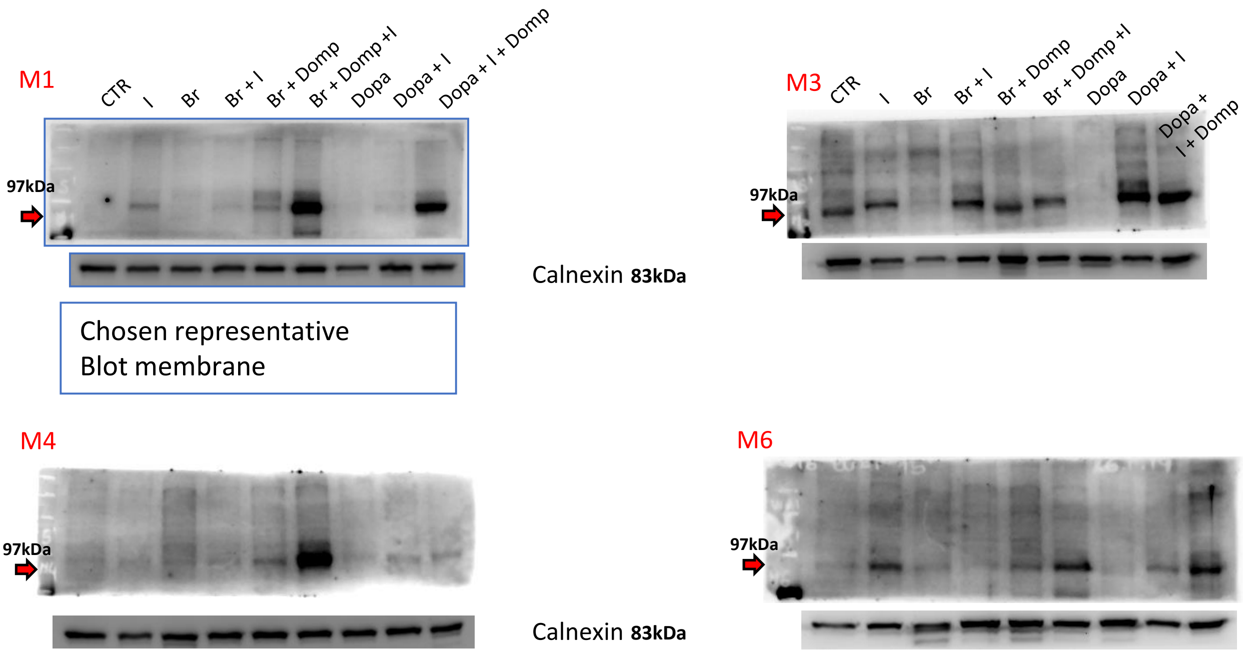

D) eWAT

InsR-Tyr972

Chosen representative
Blot membrane

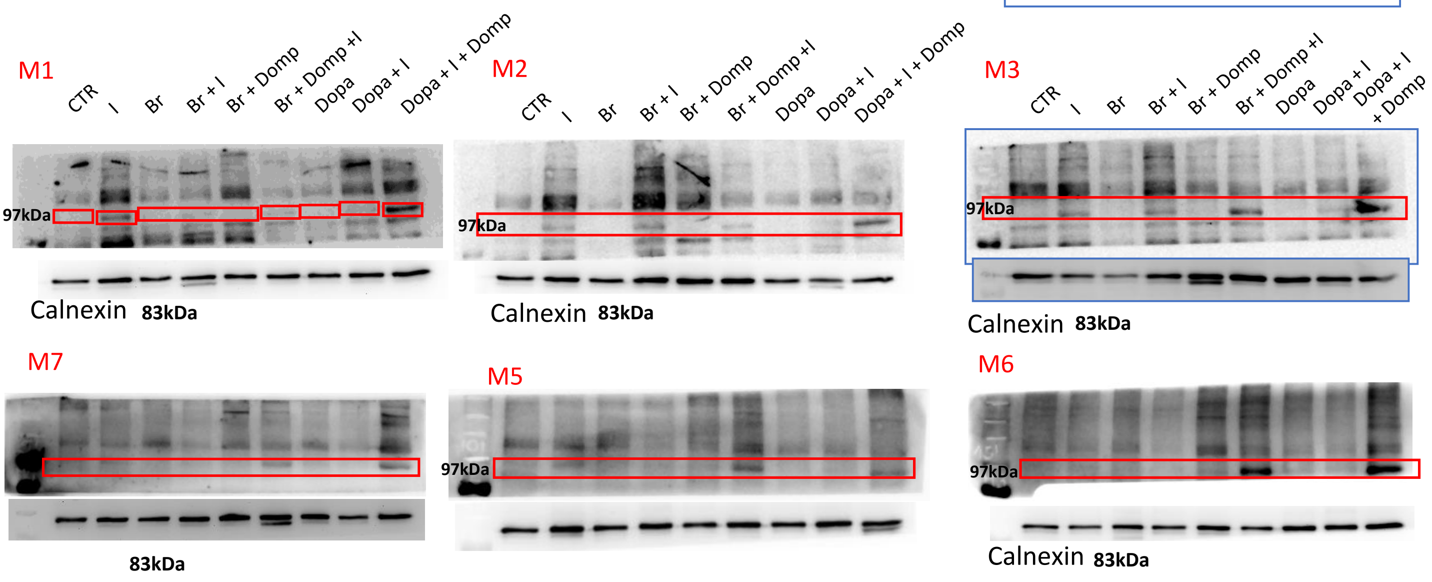

W.B. original membranes

Figure 4

A) Liver

AMPK-Thr172

Chosen representative Blot membrane

M1

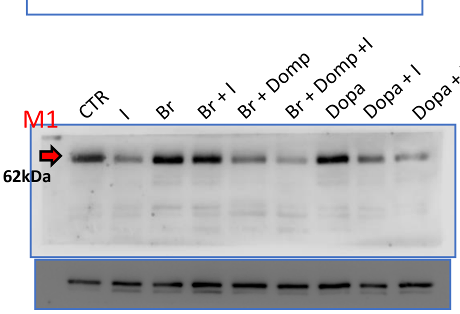

Calnexin **83kDa**

M2

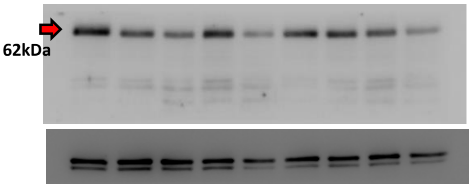

Calnexin **83kDa**

M3

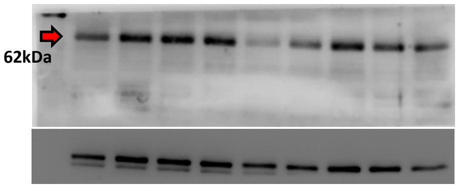

Calnexin **83kDa**

M4

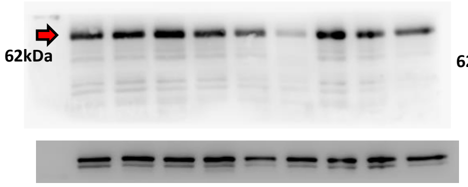

Calnexin **83kDa**

M5

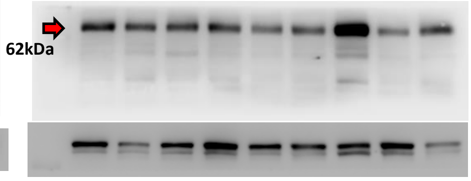

Calnexin **83kDa**

M6

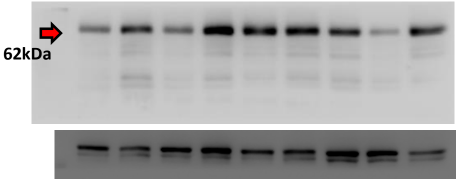

Calnexin **83kDa**

M7

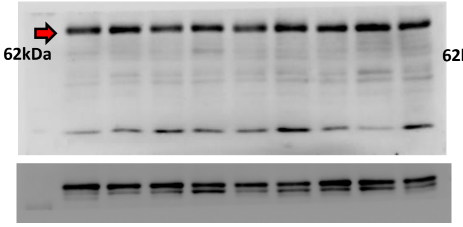

Calnexin **83kDa**

M8

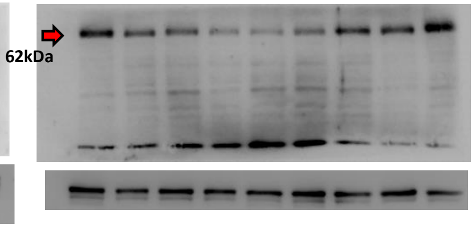

Calnexin **83kDa**

Figure 4

B) Soleus muscle

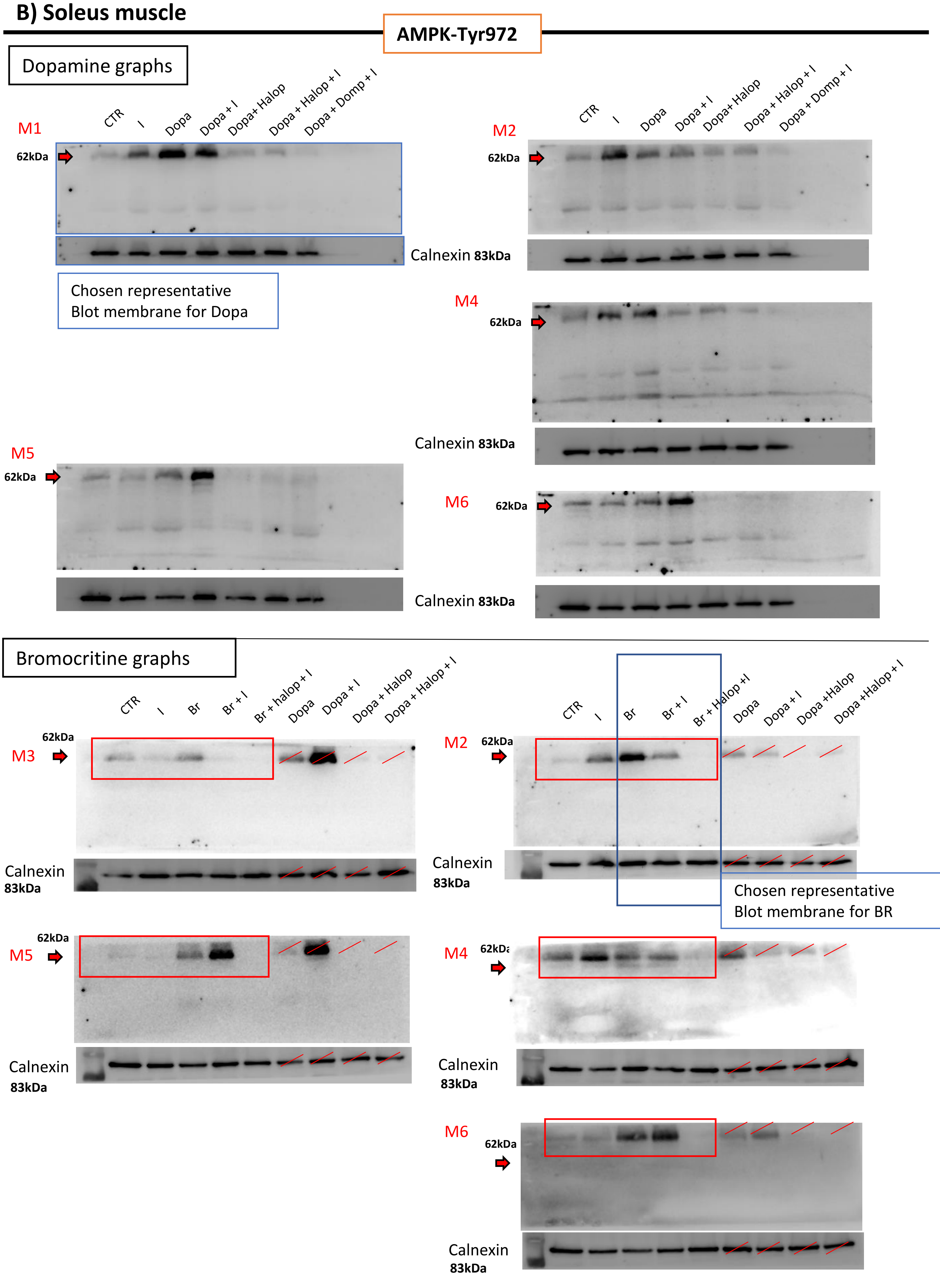

W.B. original membranes

Figure 4

C) mWAT

AMPK-Tyr972

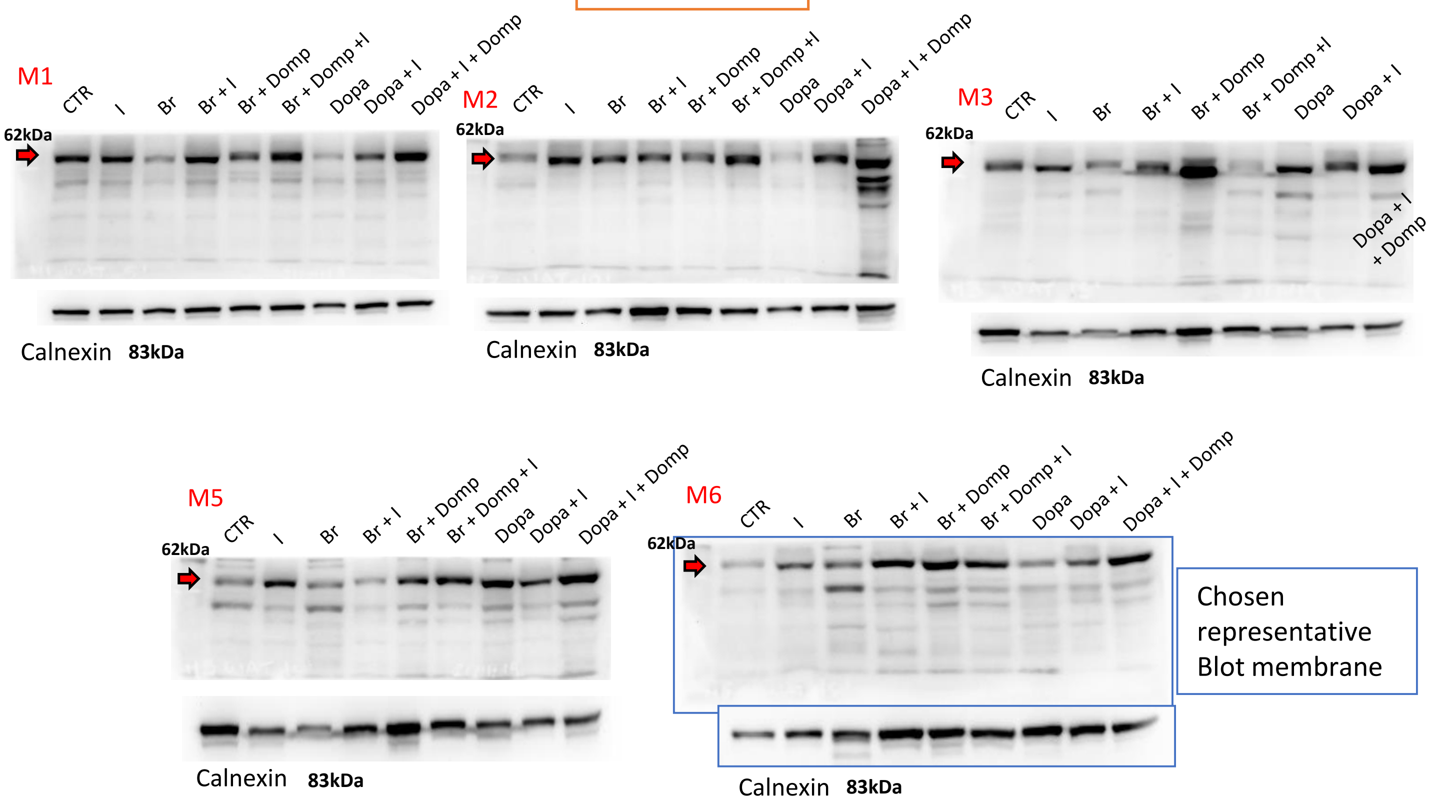

D) eWAT

AMPK-Tyr972

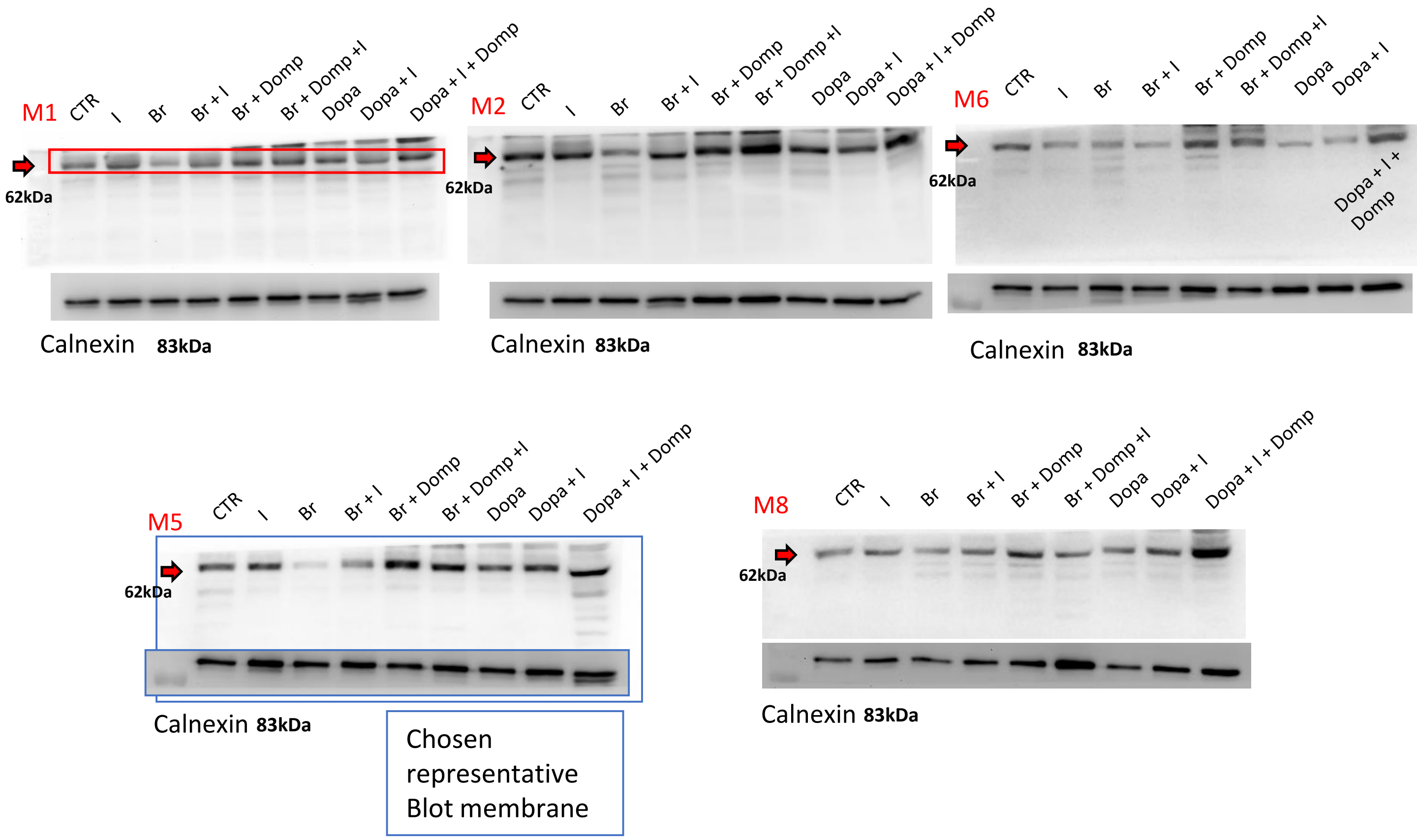

Supplement: Supplementary file 1 [file DataSheet2.PDF]
