# Supplementary figures and images for: Peripheral Dopamine Directly Acts on Insulin-Sensitive Tissues to Regulate Insulin Signaling and Metabolic Function
Source: Front Pharmacol. 2021 Sep 9;12:713418. doi: 10.3389/fphar.2021.713418 (PMC8458637; doi:10.3389/fphar.2021.713418)

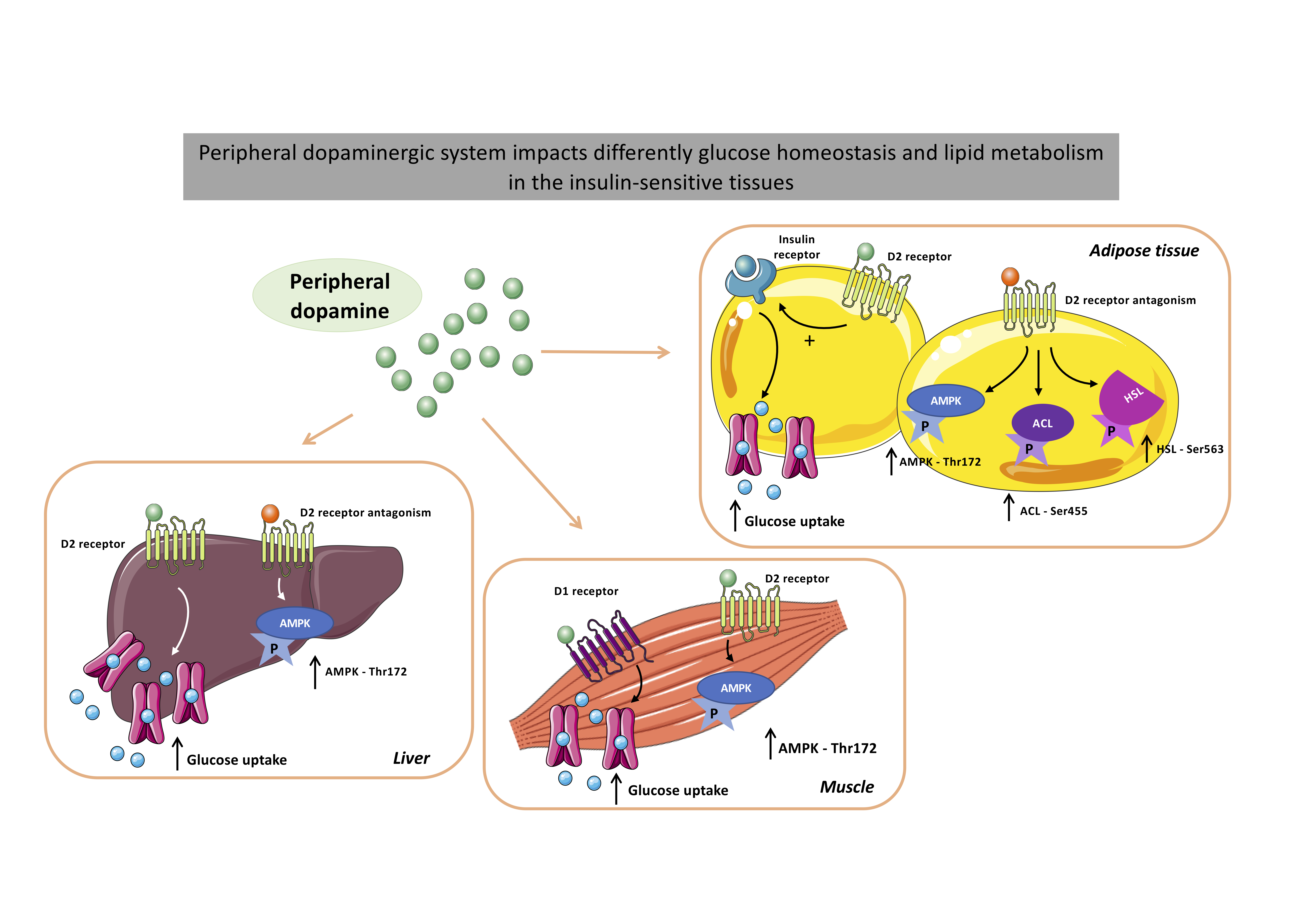

Supplement: Supplementary file 2 [file Image1.TIFF]
