# Supplementary material for: Peripheral Dopamine Directly Acts on Insulin-Sensitive Tissues to Regulate Insulin Signaling and Metabolic Function
Source: Front Pharmacol. 2021 Sep 9;12:713418. doi: 10.3389/fphar.2021.713418 (PMC8458637; doi:10.3389/fphar.2021.713418)

W.B. original membranes

Figure 5

A) mWAT

ATK-Ser473

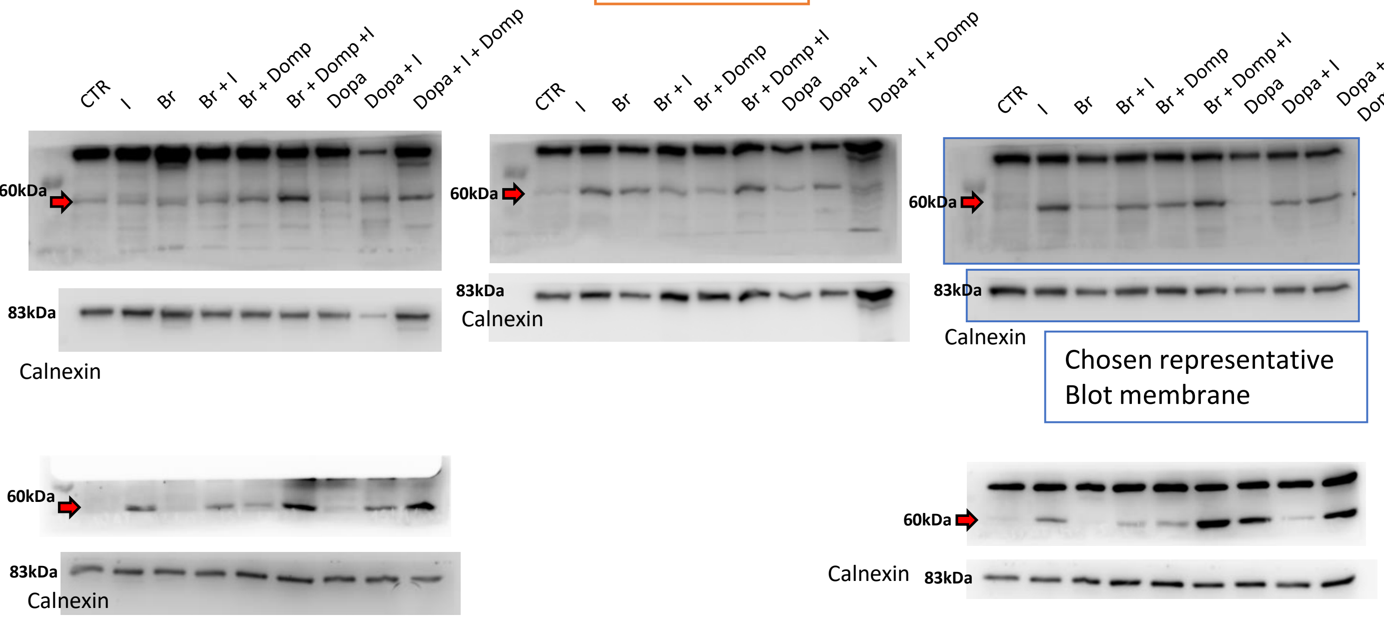

B) eWAT

ATK-Ser473

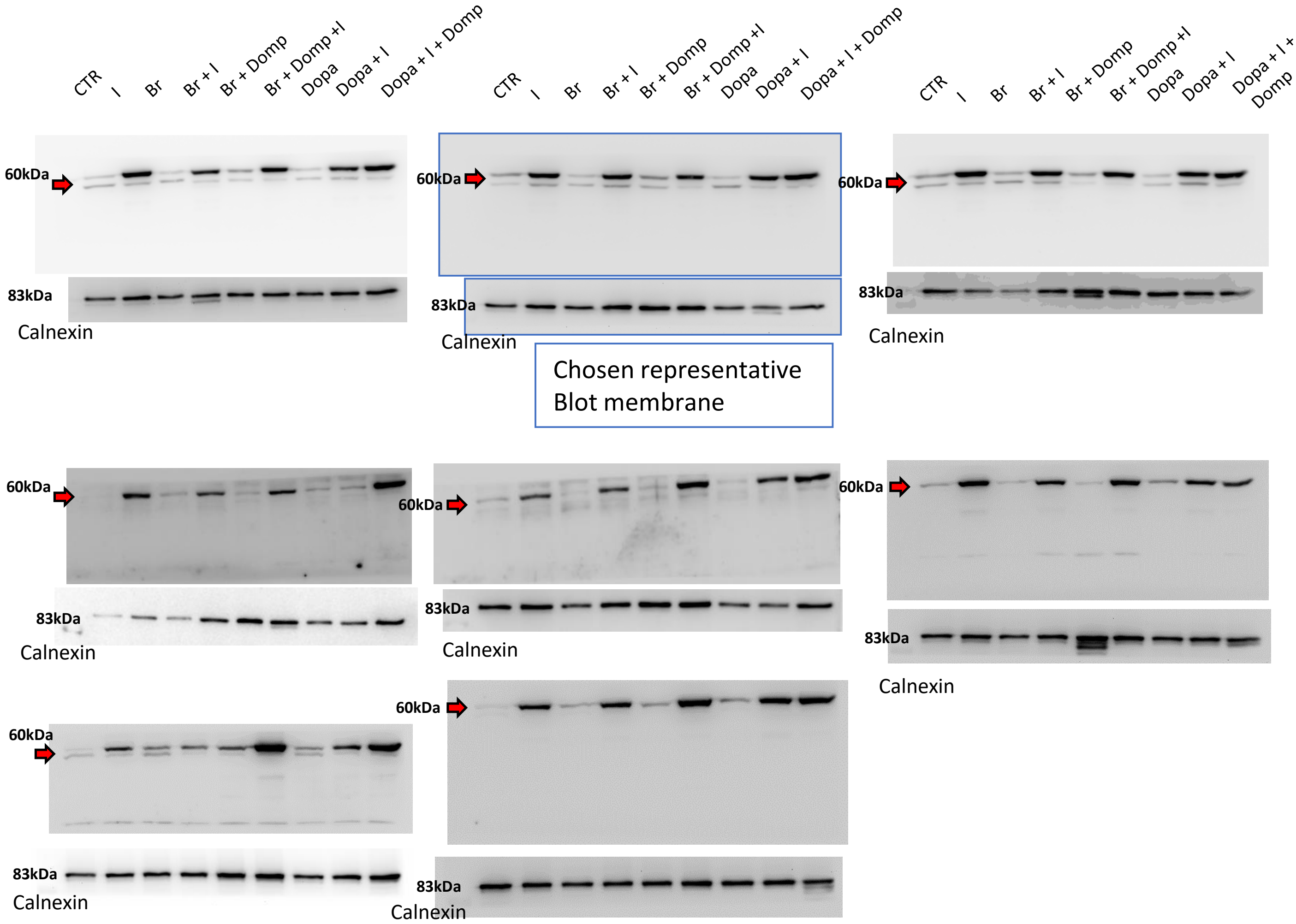

Figure 5

W.B. original membranes

C) mWAT

HSL-Ser563

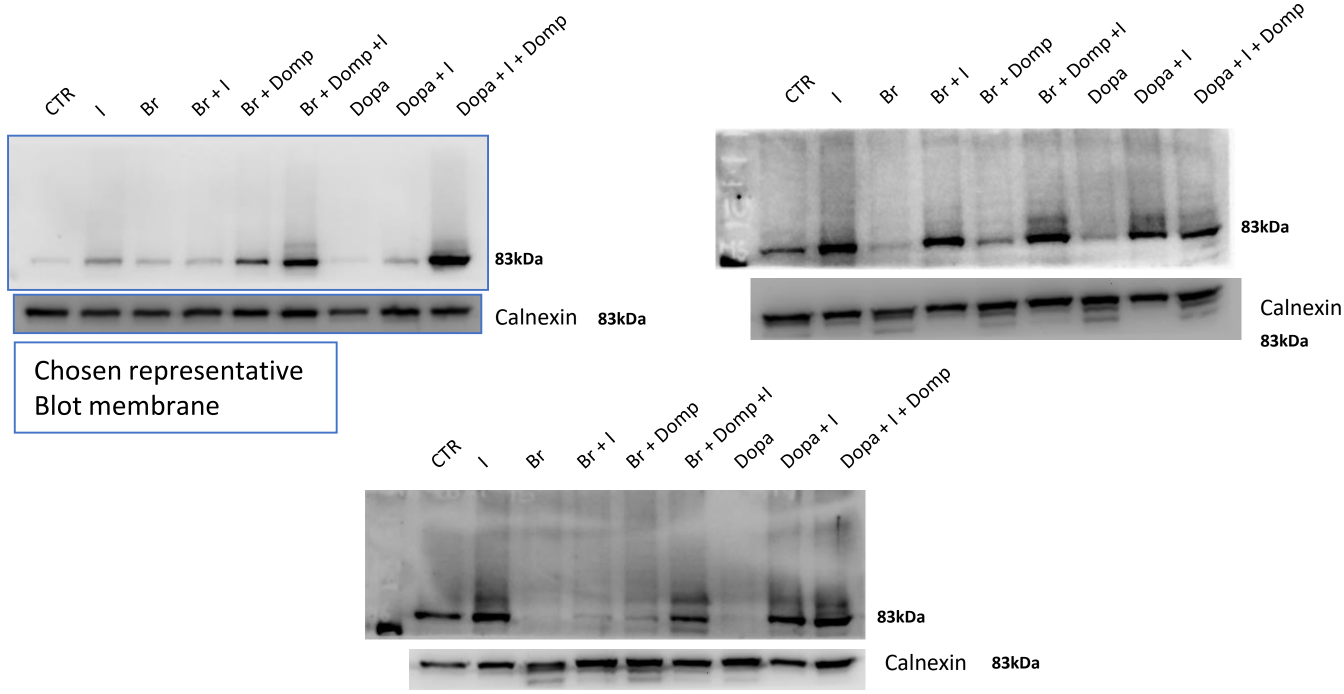

D) eWAT

HSL-Ser563

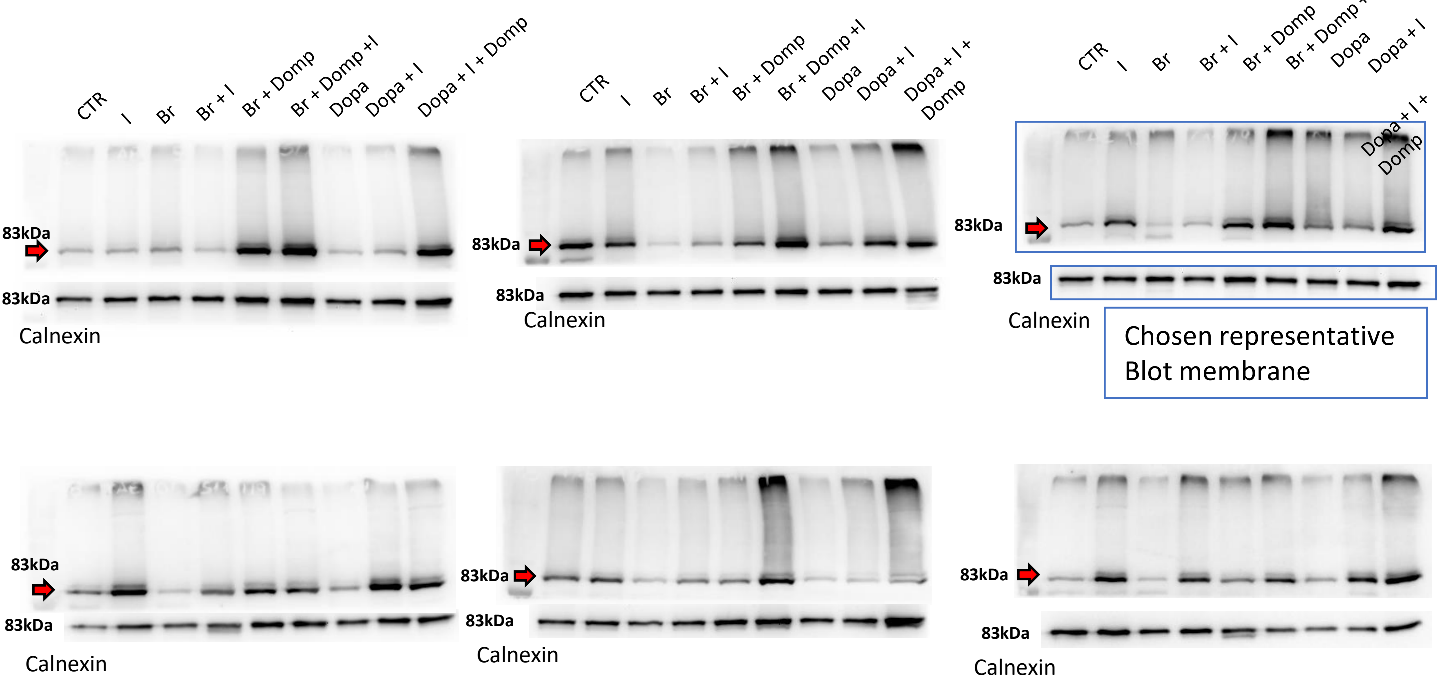

W.B. original membranes

Figure 5

E) mWAT

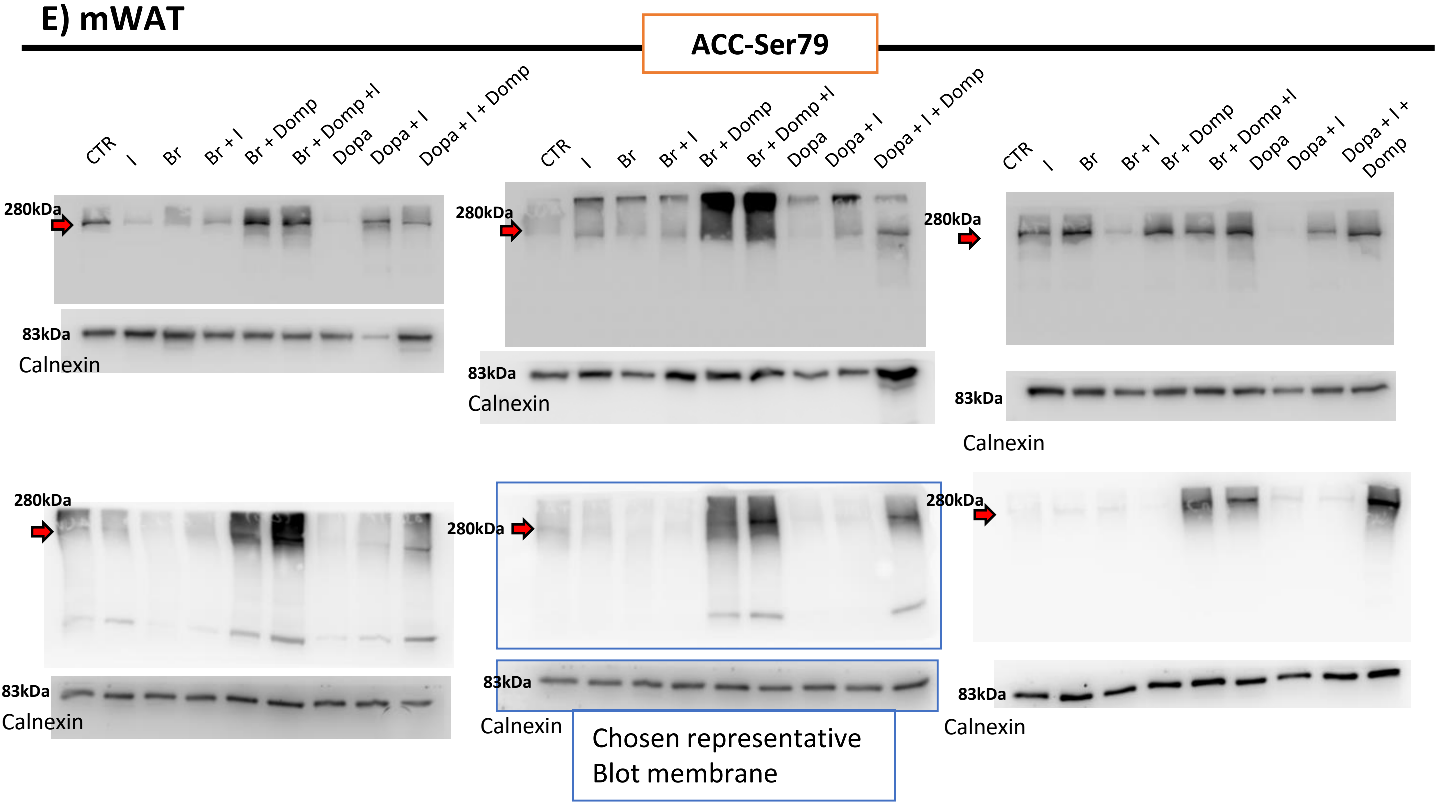

F) eWAT

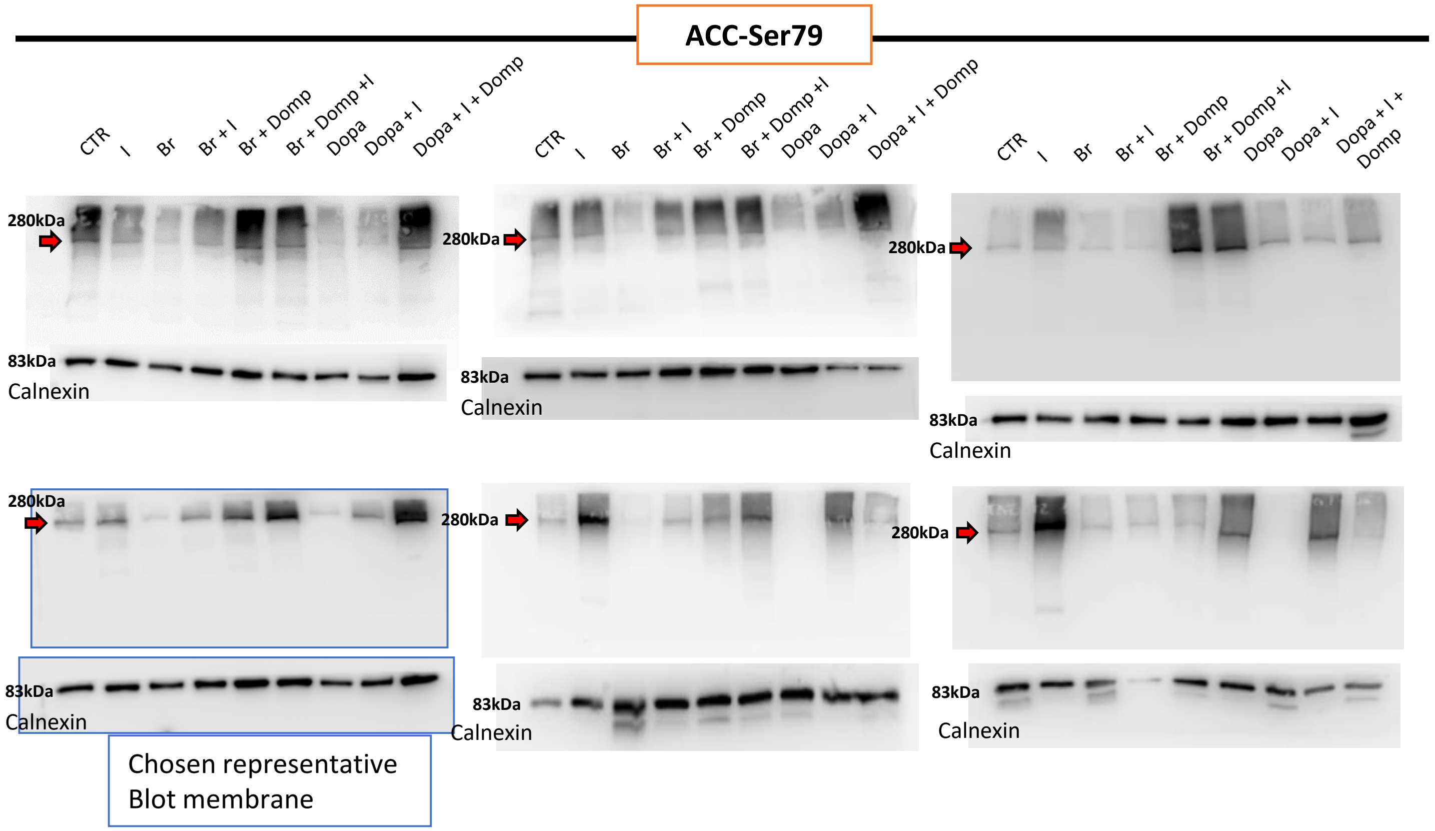

W.B. original membranes

Figure 5

mWAT

ACL-Ser455

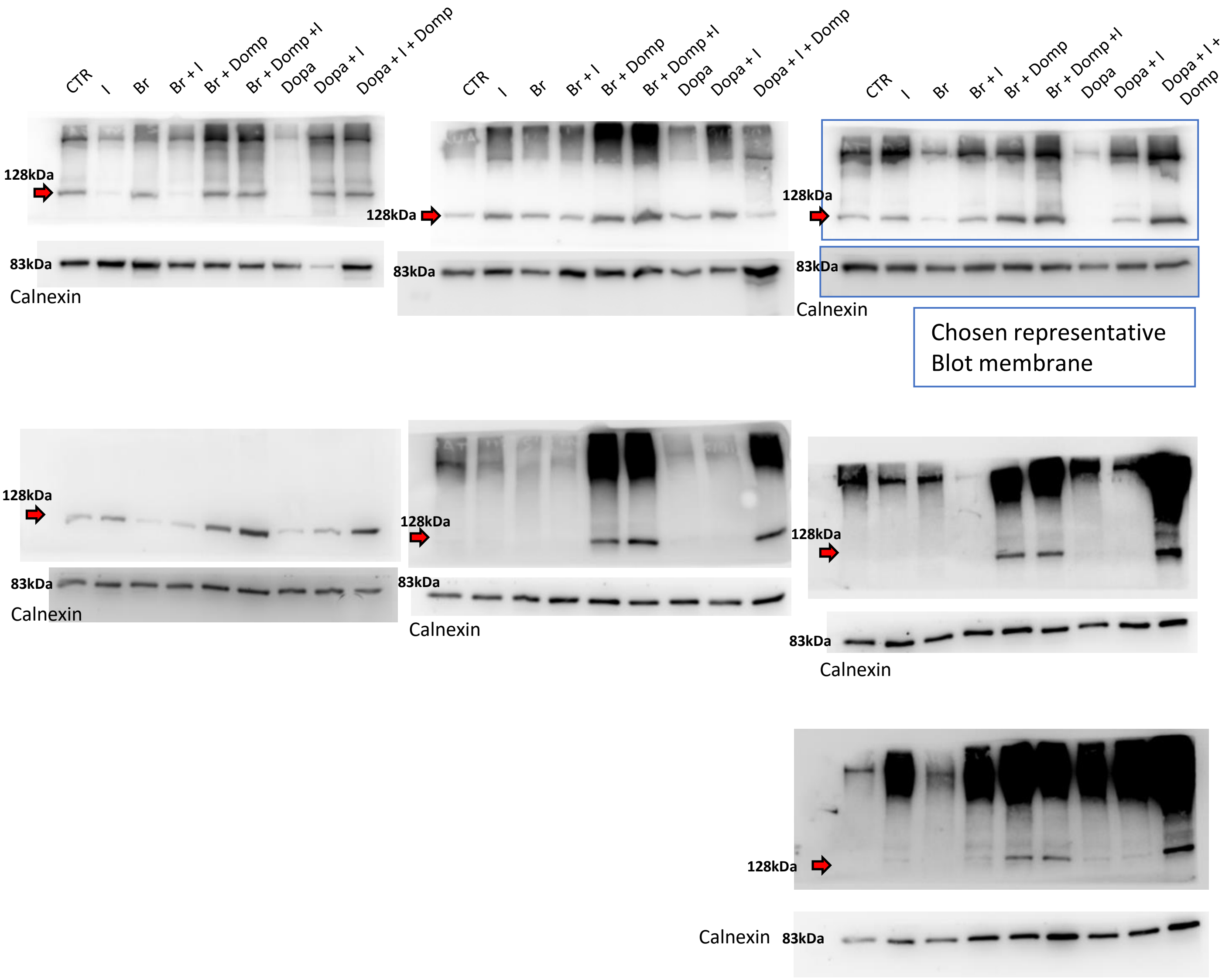

Figure 5

W.B. original membranes

H) eWAT

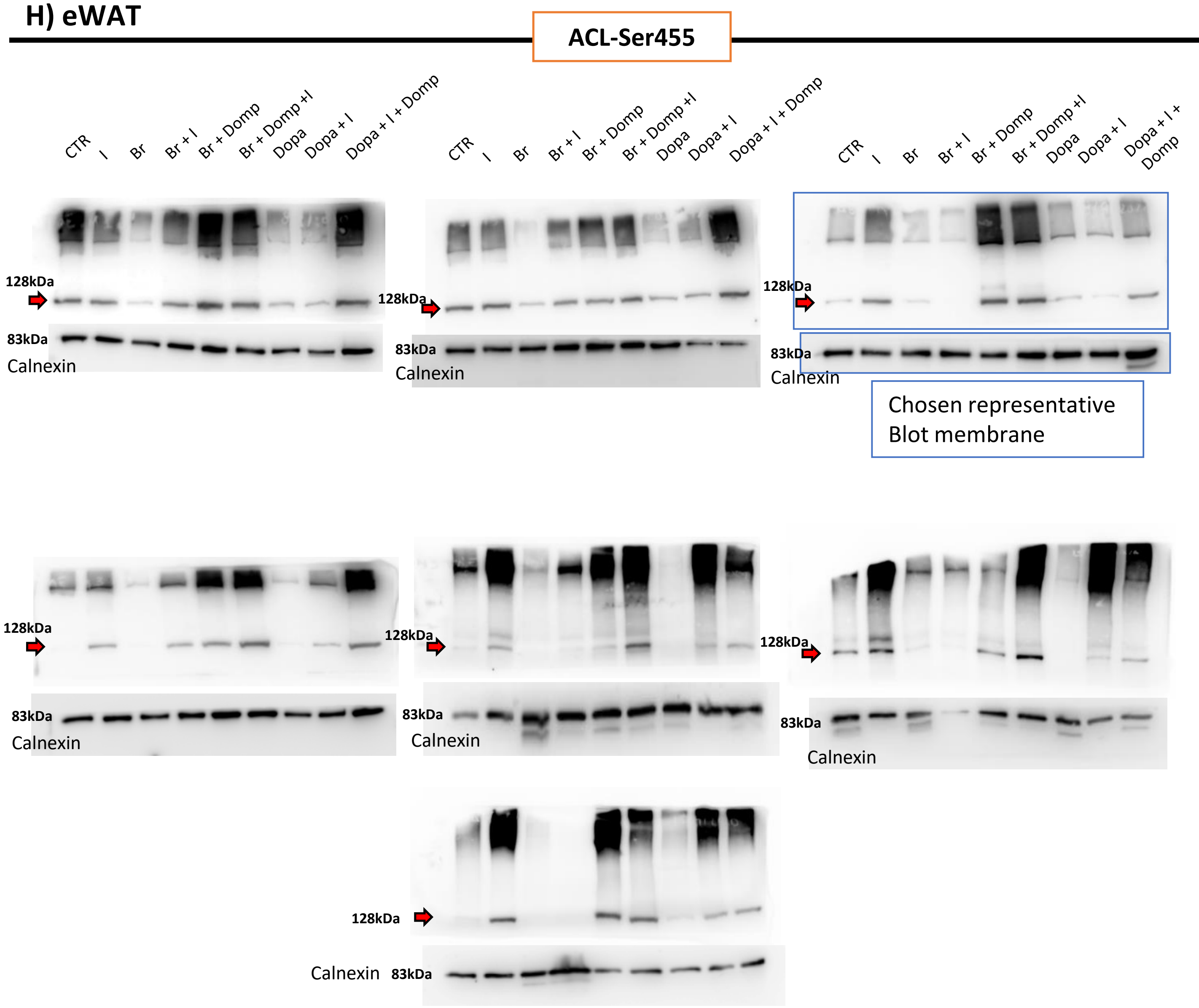

Supplement: Supplementary file 4 [file DataSheet1.PDF]
